# Architecture of the UBR4 complex, a giant E4 ligase central to eukaryotic protein quality control

**DOI:** 10.1126/science.adv9309

**Published:** 2025-08-28

**Authors:** Daniel B Grabarczyk, Julian F Ehrmann, Paul Murphy, Woo Seok Yang, Robert Kurzbauer, Lillie E Bell, Luiza Deszcz, Jana Neuhold, Alexander Schleiffer, Alexandra Shulkina, Juyeon Lee, Jin Seok Shin, Anton Meinhart, Gijs A Versteeg, Eszter Zavodszky, Hyun Kyu Song, Ramanujan S Hegde, Tim Clausen

**Affiliations:** 1 Research Institute of Molecular Pathology, Vienna BioCenter (VBC), Vienna, Austria; 2 Vienna BioCenter PhD Program, Doctoral School of the University of Vienna and Medical University of Vienna, Vienna BioCenter (VBC), Vienna, Austria; 5 Department of Life Sciences, Korea University, Seoul, South Korea; 6 Vienna Biocenter Core Facilities, Vienna BioCenter, Vienna, Austria; 7 Max Perutz Labs, Vienna Biocenter (VBC), Vienna, Austria; 8 University of Vienna, Center for Molecular Biology, Department of Microbiology, Immunobiology, and Genetics, Vienna, Austria; 9 MRC Laboratory of Molecular Biology, Cambridge, UK; 10 Medical University of Vienna, Vienna, Austria

## Abstract

Eukaryotic cells have evolved sophisticated quality control mechanisms to eliminate aggregation-prone proteins that compromise cellular health. Central to this defense is the ubiquitin-proteasome system, where UBR4 acts as an essential E4 ubiquitin ligase, amplifying degradation marks on defective proteins. Cryo-electron microscopy analysis of UBR4 in complex with its cofactors KCMF1 and CALM1 reveals a massive 1.3 MDa ring structure, featuring a central substrate-binding arena and flexibly attached catalytic units. Our structure illustrates how UBR4 binds substrate and extends K48-specific ubiquitin chains. Efficient substrate targeting depends on both pre-ubiquitination and specific N-degrons, with KCMF1 acting as a key substrate filter. The architecture of the E4 megacomplex is conserved across eukaryotes, but species-specific adaptations allow UBR4 to perform its precisely tuned quality-control function in diverse cellular environments.

Protein quality control (PQC) relies on the ubiquitin-proteasome system (UPS) to tag misfolded or mislocalized proteins for destruction before they aggregate and damage cells (1). Most ubiquitin E3 ligases recognize well-defined degrons (2), but PQC ligases must detect a broad spectrum of aberrant states (3) and often cooperate with E4 enzymes (4) that extend ubiquitin chains to reinforce the degradation signal (5, 6). Alteration of this surveillance network underlies neurodegeneration, myopathies and cancer (7, 8). Aneuploidy, for example, disrupts subunit stoichiometry, generates orphan proteins, and imposes proteotoxic stress, highlighting the importance of PQC ligases as potential therapeutic targets (9, 10).

Among the >600 human ubiquitin ligases, UBR4 plays a particularly important PQC role, being an essential and conserved E3/E4 ligase found across eukaryotes (11). UBR4 is critical for maintaining proteostasis in long-lived cells with high metabolic demands, such as neurons and muscle cells, where proteotoxic stress is particularly harmful (12–15). It recognizes several stress signatures, including mitochondrial targeting sequences (MTS) on mislocalized mitochondrial precursors, orphan proteins resulting from unassembled complexes, and aggregation-prone proteins (16–19). Additionally, UBR4 plays a key role in autophagic processes, bridging proteasomal and lysosomal degradation pathways to ensure efficient clearance of aberrant proteins (15). The diverse functions of UBR4 underscore its role as a central hub in the PQC network, capable of integrating multiple stress response pathways. Aside from teaming up with various ubiquitin ligases, UBR4 activity is linked to two specific cofactors: calcium-binding protein calmodulin (CALM1) and KCMF1 (13, 16, 17, 20, 21).

How CALM1 and KCMF1 together modulate UBR4 selectivity and its reported E4 chain-extension activity remains poorly understood. To resolve these points, we reconstituted human, nematode and plant UBR4 assemblies, defined their architectures and characterised their degradation labelling function, providing a comparative framework for UBR4’s central role in PQC and disease.

## Human UBR4 assembles a ring-shaped ubiquitination arena

We reconstituted the human E4 complex by co-expressing UBR4, KCMF1 and CALM1 in insect cells and purifying the ternary complex via a tag on UBR4. Mass photometry revealed that the isolated complex had a molecular weight of approximately 1.5 MDa consistent with a dimer of heterotrimers (Fig. S1A).

**Fig. 1:**
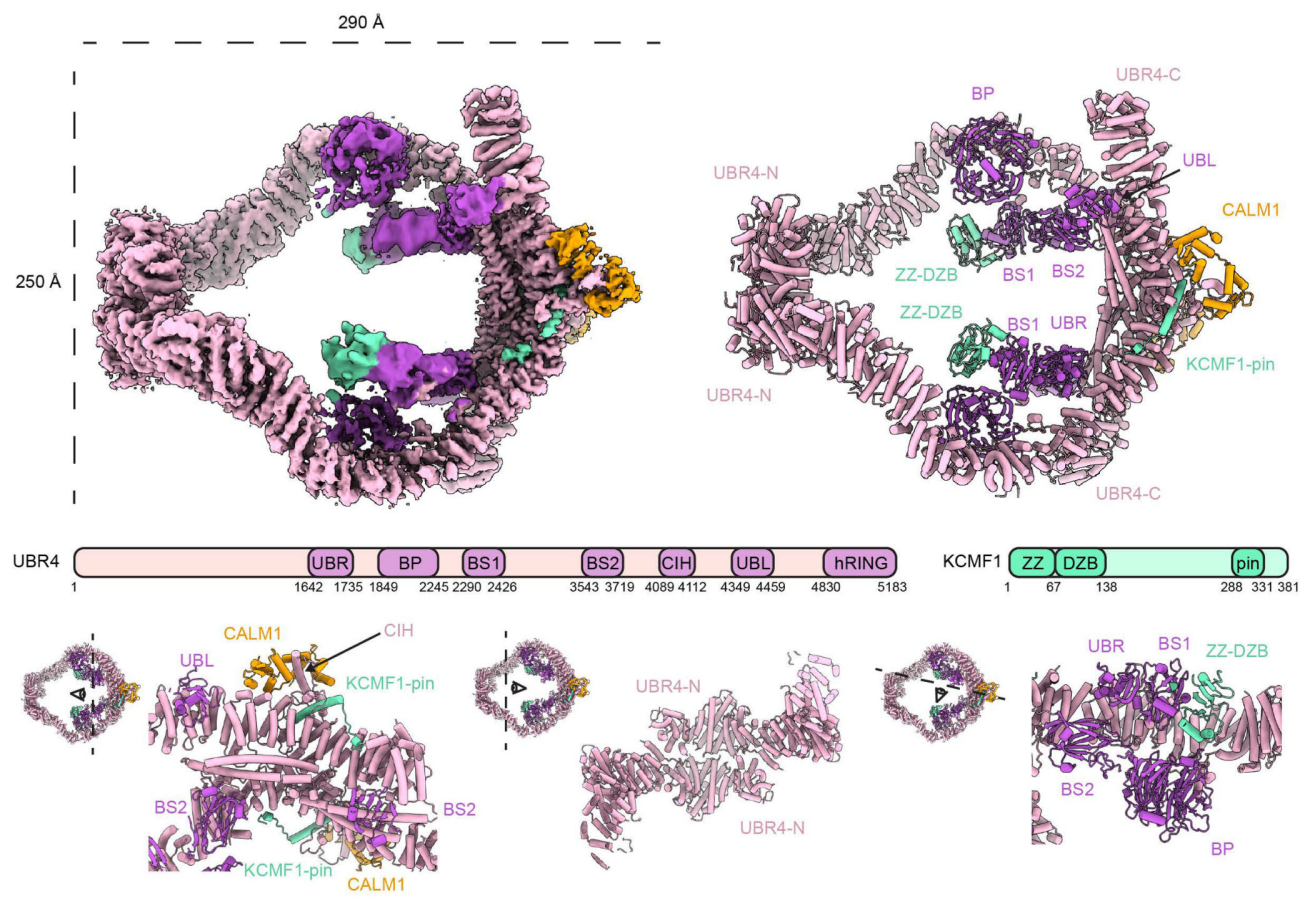
Architecture of the human UBR4 complex. Composite cryo-EM density map and model colored by protein component and domain as indicated. A schematic domain architecture of UBR4 and KCMF1 is shown below. Under this are detailed views of structural features.

Focused cryo-EM classification and refinement yielded density maps that resolved all folded regions of the human UBR4 complex (HsUBR4_2_/KCMF1_2_/CALM1_2_) (Fig. 1, Fig. S2, Fig. S3, Fig. S4), except the flexible C-terminal extension bearing the catalytic hemi-RING E3 module (22). Two UBR4 molecules dimerize through two remote, structurally different interfaces to form a large ring-shaped assembly (Fig. 1). The first interface is composed by the N-terminal domain, involving extensive contacts between adjacent Armadillo repeats. The second, larger interface is formed by Armadillo repeats in the C-terminal portion of UBR4, with laterally aligned helices sealing this contact. CALM1 and KCMF1 bind at this C-terminal interface, as predicted for the trimeric sub-complex (16). The C-terminal helix of KCMF1 inserts like a pin into a hole in the UBR4 Armadillo repeat and is covered by a small lid insertion within this region, which also mediates CALM1 binding. This arrangement implies that the proper folding of the Armadillo repeat scaffold and its associated lid depends on KCMF1. Indeed, co-expressing UBR4 with a KCMF1 variant having a mutated pin helix (L318A/F319A/V320S) yields monomeric UBR4 protein (Fig. S1B). CALM1, known to mediate calcium regulation of target proteins, engages UBR4 in a canonical manner, with a long hydrophobic CALM1-interacting helix (CIH) of UBR4 docking into the cleft between its two lobes (Fig. 1) (23). Although the C-lobe of CALM1 adopts a calcium-bound conformation (Fig. S1C), treatment with the calcium-chelator EGTA did not affect the CALM1 occupancy, suggesting that the cofactor is stably bound rather than acting as a reversible regulator (Fig. S2). Still, its precise function in the UBR4 complex needs to be discovered.

The structural motifs that constitute the ring-like scaffold of the E4 complex were well defined by EM density, but the functional domains lining the inner cavity or extending outward into the periphery displayed high flexibility, as reflected by their lower local resolution (Fig. S1D, Fig. S2, Fig. S4). The largest intrusion into the E4 cavity is a multidomain appendage formed by the beta-propeller (BP), the UBR box and the beta-sandwich 1 (BS1) of UBR4 together with the N-terminal zinc binding domains of KCMF1 (Fig. 1). A second appendix protrudes from the C-terminal dimerization region, where a zinc finger domain positions another beta-sandwich (BS2) and two associated zinc fingers near the central cavity. Finally, an extension formed by the C-terminal Armadillo repeats projects outwards from the UBR4 arena. This extension houses a ubiquitin-like (UBL) domain and a flexibly tethered hemi-RING module, the catalytic E4 domain, which was unresolved in the EM map.

## Ubiquitin K48 chain extension within the UBR4 ring

**Fig. 2:**
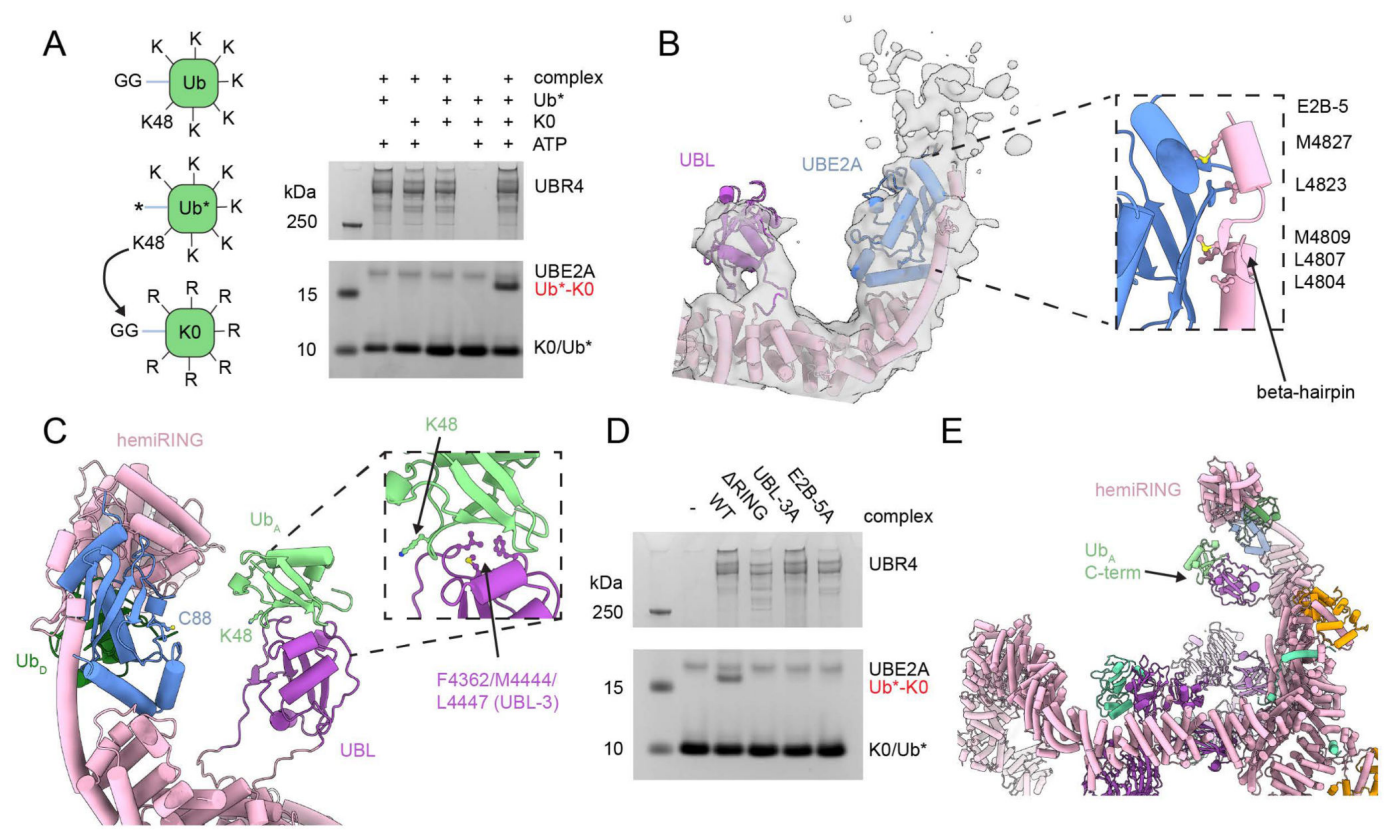
Mechanism of ubiquitin chain extension by the UBR4 complex **(A)** E4 ligase assay showing formation of K0-Ub-Ub* di-ubiquitin from K0-Ub and Ub* catalysed by 200 nM human UBR4 complex when all components of the assay are added for 45 minutes. A schematic explaining the assay and the two ubiquitin components is shown to the left. **(B)** Cryo-EM density map and model of the structure of human UBR4 complex obtained in the presence of UBE2A. An inset shows a detailed view of the interaction highlighting hydrophobic residues on the E2-binding helix of UBR4 which are mutated in the E2B-5A UBR4 complex variant. **(C)** Model of the E4 transfer state using our structure overlaid with a crystal structure of the hemiRING-UBE2A complex (PDB pdb_00008BTL) and the cryo-EM structure of the yeast UBR1-UBE2A-Ub_D_ complex (PDB pdb_00007MEX), both aligned on UBE2A, and an AlphaFold3 model of the UBL-Ub interaction (Fig. S5A) aligned on the UBL domain. The three hydrophobic residues on the UBL domain which interact with ubiquitin and are mutated in the UBL-3A variant are indicated. **(D)** E4 ligase assay as in panel **A** with 100 nM of the indicated variant complexes for 45 minutes. **(E)** Zoomed out image of the E4 transfer state model showing the position of the C-terminal tail of Ub relative to the UBR4 arena.

To investigate how the distinct functional motifs of the UBR4 complex support ubiquitination, we devised an E4 assay monitoring the conjugation of the two ubiquitin variants Ub* and Ub-K0 (Fig. 2A). Ub* (ubiquitin-ΔGG) lacks the C-terminal di-glycine motif required for ubiquitin transfer, whereas Ub-K0 has all lysine residues mutated to arginine. This setup enabled us to follow a single ubiquitination event, where Ub-K0 is transferred onto Ub*, and then no further reaction can occur. As expected, a Ub-K0-Ub* band occurred only in the presence of both ubiquitin variants and UBR4 (Fig. 2A), offering a specific readout of E4 activity, ideal for mutational analyses and systematic substrate screens.

We had previously shown that UBR4 only ubiquitinates orphan proteins which have already been ubiquitinated by other E3 ligases (16). This E4 activity depends on the UBL domain which was predicted by AlphaFold3 (Fig. S5A) (24) to bind ubiquitin and orient lysine K48 towards the E2~Ub conjugate at the hemi-RING, favoring K48-linked chain extension on pre-ubiquitinated substrates (16). In line with this, formation of free ubiquitin chains by the UBR4 complex required K48 of ubiquitin (Fig. S5B). To probe the UBL/Ub interface, we mutated three residues in the predicted binding site of the UBL domain (UBL-3A). Analytical size-exclusion chromatography (Fig. S5C), which was performed with the isolated and stably folded UBL variants (Fig. S5D), showed that wild-type UBL interacted with ubiquitin, whereas the UBL-3A variant did not.

We then incubated the UBR4 complex with its cognate E2 enzyme (UBE2A) and analyzed the resulting state by cryo-EM (Fig. S6, Table S2). The reconstruction contained extra density for UBE2A, enabling us to model the E2 bound to the C-terminal extension of UBR4, despite the low resolution in this region (Fig. 2B). As predicted by AlphaFold3, the E2 is bound in a backside orientation, stabilized by a specific beta-hairpin structure inserted within an extended helix of the C-terminal protrusion of UBR4 (16). The largely hydrophobic interface covers two sides of UBE2A yet leaves its canonical RING-binding face free for hemi-RING engagement. As a consequence, the E2 becomes precisely positioned near the UBL domain at the narrow constriction site of the substrate binding arena. In our cryo-EM map, the catalytic hemi-RING is not engaged in direct contacts with UBE2A, but appears as diffuse density above the E2 (Fig. S6), consistent with its reported low affinity for UBE2A (22). We therefore modelled the ubiquitin transfer state by fitting the UBE2A-UBR4(hemi-RING) crystal structure (22) onto UBE2A of our cryo-EM model (Fig. 2C). Further, we used the yeast UBR1-UBE2A-Ub structure to model the donor ubiquitin (25), whereas the acceptor ubiquitin was placed using the AlphaFold3 predicted UBL/UB complex (Fig. S5A). In this model, guided by our cryo-EM structure, UBE2A and UBL domain align the acceptor ubiquitin so that its K48 residue points towards the E2~Ub thioester at the hemi-RING, thus favoring K48 ubiquitin chain extension. Consistent with this putative E4 mechanism, Ub-K0–Ub* formation was abolished by the UBL-3A mutation, deletion of the hemi-RING (ΔRING), or by disrupting the backside interface of UBE2A (E2B-5A) (Fig. 2D). In native substrates, the UBL-bound ubiquitin would be covalently attached through its C-terminus to a lysine residue in the target protein. As the ubiquitin C-terminus points into the central cavity of the UBR4 complex, potential substrate recognition sites should reside within this region (Fig. 2E).

## Selection of Ub-marked proteins to be degraded

**Fig. 3:**
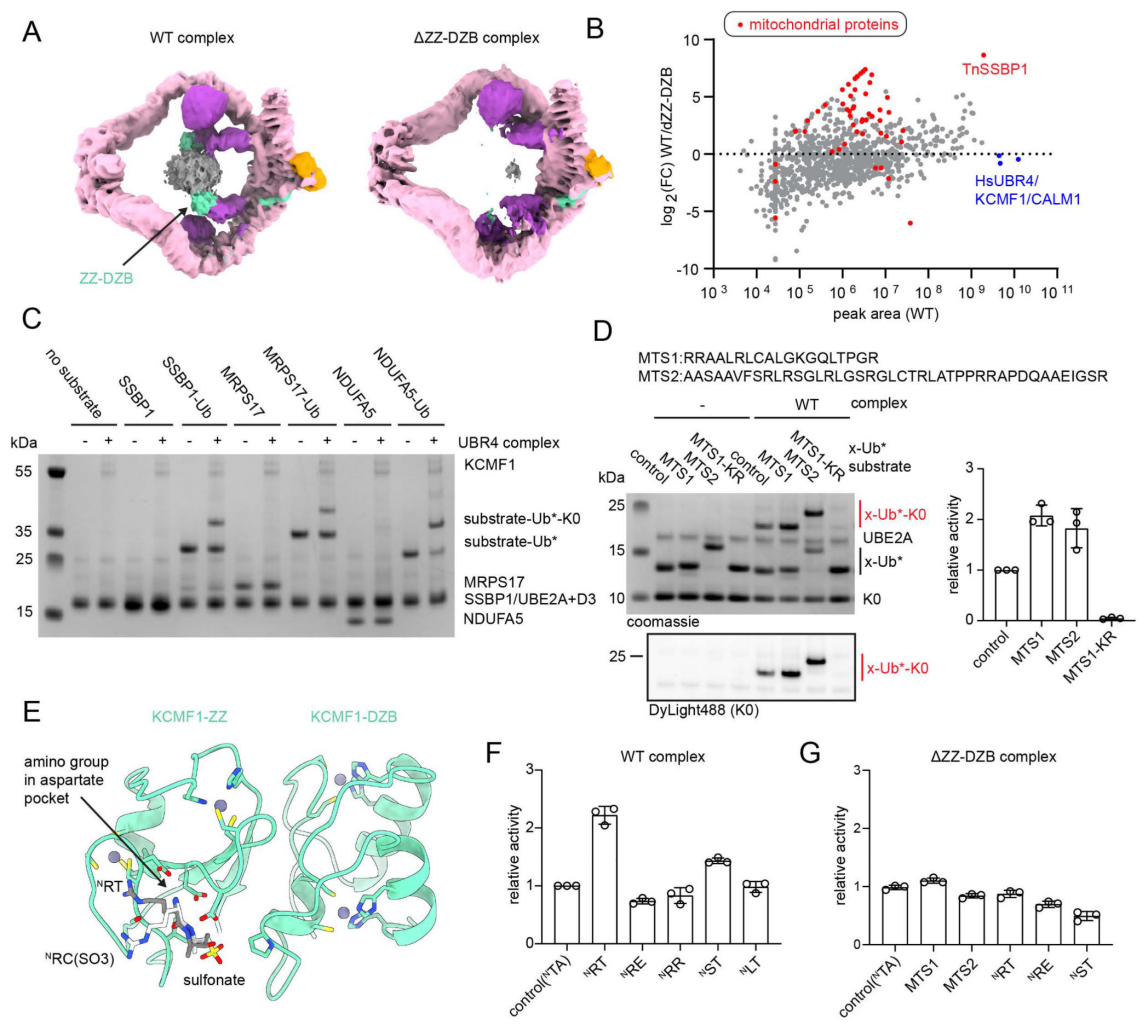
KCMF1 mediates selection of UBR4 substrates. **(A)** On the left, a cryo-EM map of the human UBR4 complex structure refined with a global mask showing fuzzy central substrate density and on the right, the ΔZZ-DZB UBR4 complex refined with the same approach. **(B)** MS analysis of insect cell proteins co-purified with the WT and ΔZZ-DZB UBR4 complexes where peak intensity in the WT is plotted against log_2_(fold change) between the two datasets to identify proteins which are present in the WT but not the ΔZZ-DZB UBR4 complex. The core complex components are coloured blue and mitochondrial proteins are coloured red. (**C**) Ubiquitination assay using 200 nM UBR4 complex, UBE2A, UBE2D3 and K0-Ub for 30 minutes in the presence of 2 μM of the indicated recombinantly purified insect cell proteins with or without Ub* fused by a linker to the C-terminus. (**D**) E4 ligase assay with 200 nM of the human UBR4 complexes for 30 minutes with the indicated MTS sequence, or control with no MTS, fused via a linker to the N-terminus of Ub*. KR indicates a K48R mutation in the fused Ub*. Reactions contained 1:10 DyLight488 K0-Ub which was used to quantify product bands relative to the control substrate and plot these on the right (+/- SD). **(E)** Two overlaid crystal structures of the KCMF1^ZZ+DZB(△linker)^ domain with either an N-RC(SO_3_)K peptide or an N-RTGG peptide. Only the two N-terminal residues are shown for clarity along with important residues on KCMF1 shown in stick representation. **(F-G)** Quantified E4 ligase assays (+/- SD) as in panel **D** with the indicated substrates for (**F**) 200 nM WT UBR4 complex for 30 minutes and (**G**) 200 nM ΔZZ-DZB UBR4 complex for 15 minutes. Assay gels are shown in Fig. S12A,B.

The UBR4 complex has mobile E4 catalytic arms on top of a huge substrate binding arena. This organization is reminiscent of the giant ubiquitin ligases BIRC6, HUWE1 and UBR5, which use an array of receptor domains to target diverse substrates (Fig. S7A) (26–32). While analyzing our cryo-EM data, we noted additional low-resolution density inside the central cavity of the UBR4 complex (Fig. 3A). As we could assign all ordered domains of the UBR4 complex, we suspected that this extra density represents a copurified substrate bound in the inner cavity, visualizing the mechanism of substrate recognition. The density was connected to the E4 by the ZZ-DZB motifs of KCMF1, hinting that these domains act as substrate receptors. To test this idea, we reconstructed an UBR4 complex lacking ZZ-DZB and determined its cryo-EM structure using the same workflow as for the wild-type complex (Fig. S8, Table S2). In the resulting map, the ZZ-DZB domains were absent as was the connected extra density (Fig. 3A). To identify captured substrates, we performed an MS analysis searching for proteins that co-purified with wild-type E4 but were lost in the ΔZZ-DZB sample (Fig. 3B). Single stranded DNA binding protein (SSBP1), a mitochondrial protein previously reported to bind to UBR4/KCMF1 in human cells (13), was the most enriched protein in the wild-type complex as compared to the ΔZZ-DZB deletion. Many other proteins preferentially retained in wild-type UBR4 were mitochondrial, consistent with reports that UBR4 participates in mitochondrial PQC (13, 17). By contrast, deleting other putative receptors such as the BS2 or UBR domains did not enrich obvious targets (Fig. S7B).

The UBR4 complex has been implicated in targeting unimported mitochondrial precursors that still carry their unprocessed mitochondrial targeting sequence (MTS) (13, 17). To ask whether the insect cell (*Trichoplusia ni)* proteins copurifying with the UBR4 complex retained an MTS, we applied a tailored MS analysis mapping their N-terminal sequences (Fig. S7C). Some hits, such as SSBP1 and ACADS, had a processed MTS whereas others, such as NDUFA5 and MRPS17, contained an intact signal sequence. To test potential substrates in ubiquitination assays, we produced recombinant variants of the identified proteins with the detected N-termini. We generated each variant with and without a C-terminally fused Ub* to explore whether the UBR4 complex can extend chains on pre-ubiquitinated substrates (16, 33) or relies on KCMF1 as priming E3 ligase (34). To simplify the read-out, we used K0 ubiquitin which prevents chain formation. Robust ubiquitination was only observed for Ub*-fused TnSSBP1, TnMRPS17 and TnNDUFA5 but not for their unfused counterparts (Fig. 3C), indicating that the UBR4 complex acts as a chain extending E4 ligase, not a priming ligase. Mutating individual lysines on ubiquitin showed that generated chains were K48-linked (Fig. S9A). In a control experiment, we used HUWE1 as priming E3 ligase and monitored the ubiquitination of TnNDUFA5, predicted to be a good HUWE1 substrate (28). In contrast to KCMF1, HUWE1 was able to initiate ubiquitin chains on TnNDUFA5 (Fig. S9B), which were further extended by the UBR4 complex (Fig. S9C). These data suggest that KCMF1 does not function as priming E3 ligase in the UBR4 complex. Consistently, KCMF1 lacks a known E3 domain (Fig. S9D), did not interact with any E2 in an AlphaFold pulldown screen (Fig. S9E), and did not catalyze ubiquitination with any E2 we tested (Fig. S10).

To pinpoint substrate features recognized by the UBR4 complex, we adapted our E4 assay. Appending two model MTS sequences via a linker onto the N-terminus of Ub* enhanced K48 chain extension compared to the MTS-free control (Fig. 3D). Thus, substrate features beyond ubiquitin itself can enhance recruitment to the UBR4 complex. Because the ZZ-DZB motif of KCMF1 has been implicated in binding Arg N-degrons (35), we measured the binding of the isolated ZZ-DZB domain to synthetic peptides by isothermal titration calorimetry (ITC) (Fig. S11A). No interaction was observed for the reported ligand Arg1-Cys2sulfonate, ^N^RC(SO3) and, consistently, a co-crystal structure of the ZZ-DZB:peptide complex showed both R1 and C(SO3)2 sidechains protruding out of the binding pocket (Fig. 3E, Fig. S11B). Screening additional peptides revealed tight binding to an ^N^RT motif, and the co-crystal structure captured specific contacts with both the T2 side chain and the N-terminal amino group (Fig. 3E, Fig. S11C, Table S3). ITC binding studies showed that the second residue is more important than the first, as an ^N^LT peptide also interacted with the ZZ-DZB domains but ^N^RL, ^N^RR or ^N^RA peptides did not (Fig. S11A). A threonine at position 2 is characteristic of many processed mitochondrial proteins, for example identified TnSSBP1 (^N^ST) and TnACADS (^N^FT) and previously co-purified human proteins such as ABHD10 (^N^KT), SARS2 (^N^TT), ACOT9 (^N^LT) (13) as well as the Dengue virus protein NS5 (^N^GT) binding UBR4 (36). In agreement with these data, E4 assays showed stronger chain extension on a substrate with an ^N^RT motif compared to other N-termini (Fig. 3F, Fig. S12A). Determining whether this specificity was due to the ZZ-DZB motif was complicated as the deletion produced more free chains for unknown reasons (Fig. S9F). Nevertheless, its specificity profile differed from wild-type, showing little preference among control, MTS or N-degron containing constructs but still reduced activity on ^N^RE and ^N^ST termini (Fig. 3G, Fig. S12B). Together, these data identify the ZZ-DZB as a major substrate receptor, and other domains in the E4 arena might finetune specificity.

## Evolutionary conservation of the UBR4 ubiquitination arena

UBR4 plays important roles in various eukaryotes, including *Arabidopsis thaliana* (37, 38) and *Caenorhabditis elegans* (39) yet its partnership with KCMF1 and calmodulin has not been demonstrated outside animals. To test whether the human E4 architecture and mechanism represents a universal PQC solution, we first reconstituted the *C. elegans* UBR4 complex by co-expressing CeUBR4, CeKCMF1 (ZK652.6) and CeCALM. The purified complex had a molecular weight of 1.1 MDa consistent with a dimer of trimers (Fig. S13A). Activity assays showed that the CeUBR4 complex preferred MTS-Ub* substrates over Ub* alone but showed only weak selectivity for the ^N^RT-Ub* degron (Fig. S13B).

**Fig. 4:**
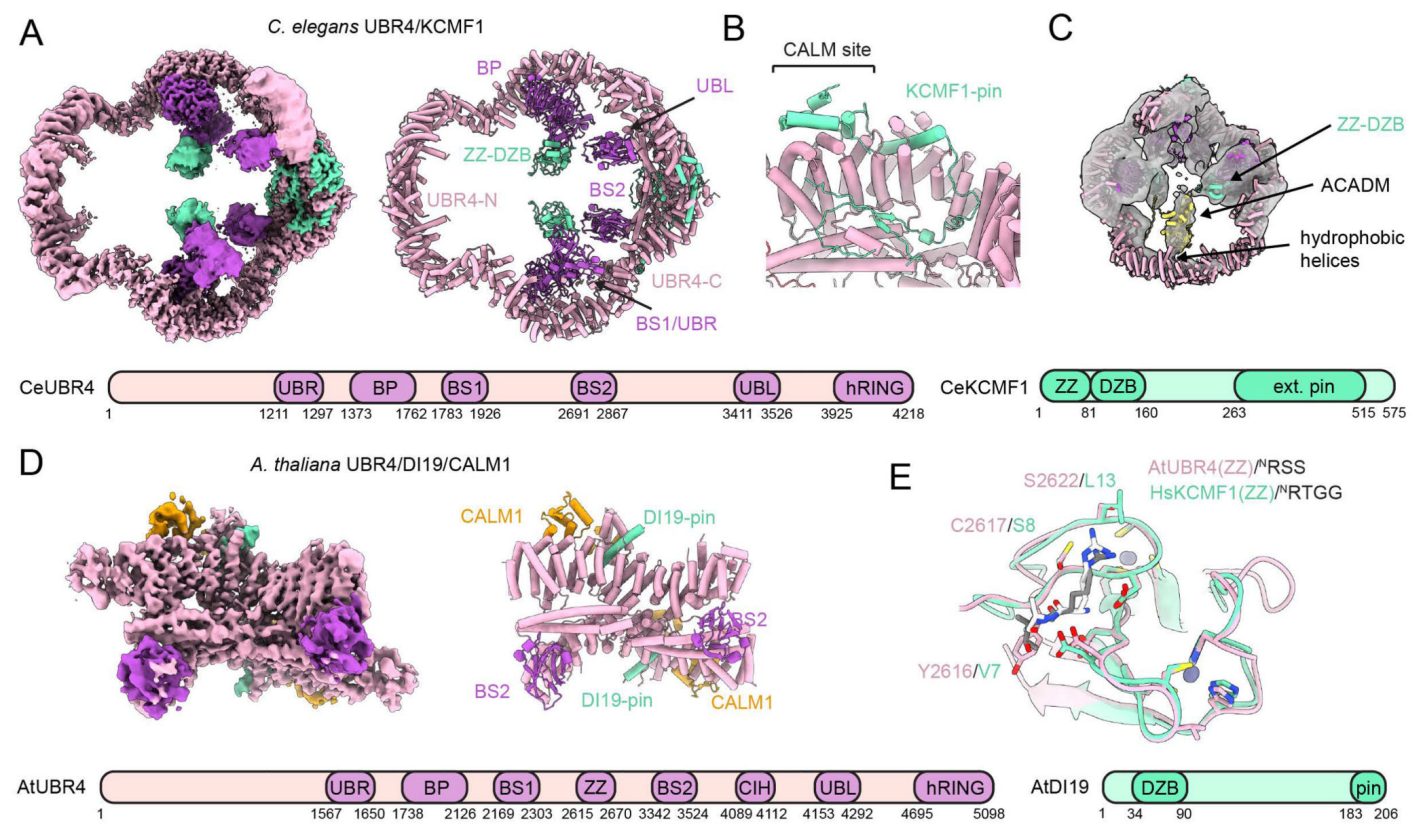
Evolutionary conservation of the UBR4 complex **(A)** Cryo-EM composite map and model of the *C. elegans* UBR4 complex coloured by subunit and domain as indicated. The domain architecture is illustrated below. **(B)** Detailed view of the extended interface of CeKCMF1 with CeUBR4. (**C**) Low resolution cryo-EM map of the CeUBR4 complex from a global 3D classification (Fig. S14) showing a bound substrate. An AlphaFold3 model of the co-purified protein TnACADM is modelled in the density. (**D**) Cryo-EM map of the dimer interface core of the AtUBR4 complex coloured by domain and subunit as indicated. The domain architecture derived from bioinformatic comparison with the human UBR4 complex is shown below. (**E**) Crystal structure of the AtUBR4 ZZ domain in complex with an N-RSS peptide superimposed with the HsKCMF1 ZZ domain crystal structure in complex with an N-RTGG peptide. Sequence differences in the N-degron binding pocket are highlighted.

To explore structural differences, we determined the cryo-EM structure of CeUBR4 (Fig. 4A, Fig. S14, S15, S16, Table S4). Although the nematode E4 complex preserves the ring shape of the human enzyme, it displays notable adaptations, particularly in the dimerization interfaces that result in different topologies of the ring-shaped arena. Whereas the domains aligning the N-terminal ends exhibit different, unrelated scaffolds, the C-terminal interface is sealed by distinct interaction with the two co-factor proteins. Most notable, in the CeUBR4 complex, CALM1 is absent, and its binding site is filled by insertions in CeKCMF1 (Fig. 4B). The necessity of occupying this interface in the absence of CALM1 suggests a role in maintaining the structural integrity of the E4 complex. Proteomic analysis of purified CeUBR4 pointed to a distinct substrate preference, with mitochondrial medium-chain acyl-CoA dehydrogenase (ACADM) showing stronger enrichment compared to SSBP1, which is preferred by the human UBR4 complex (Fig. S13C, Table S5). In line with this finding, we observed well-defined extra density in the central arena of CeUBR4, closely resembling the elongated shape of an orphaned subunit from the tetrameric ACADM complex (Fig. 4C). According to the cryo-EM data, the bound substrate not only contacts the ZZ domain but also two long helices in the N-terminal region. These two helices, which are unique to CeUBR4, protrude from the rigid E4 core into the central cavity, exposing a series of hydrophobic residues (Fig. S13D) that may help to recognize and bind the hydrophobic surface characteristic of orphaned subunits.

The observed adaptations of the CeUBR4 complex prompted us to investigate the ancestral design of the UBR4 core further. We turned to *Arabidopsis thaliana* UBR4 (also known as BIG), which diverged from metazoan UBR4 early in eukaryotic evolution and has distinct roles in hormone signaling (37, 38). Because KCMF1 is critical for E4 assembly, we first performed an in-depth bioinformatic analysis to identify the elusive plant orthologue. Drought-induced protein 19 (DI19) emerged as the best candidate: it shows strong homology to HsKCMF1 in the C-terminal pin region (Fig. S13E), has related biological functions to AtUBR4 (37, 40) and was one of the top hits alongside AtUBR4 in an N-degron proximity labelling screen (38). Co-expression of AtUBR4, AtDI19 and AtCALM1 followed by cryo-EM analysis on the resulting complex (Fig. S17, Table S3) yielded a medium resolution map of the dimerization interface, which we could model using AlphaFold3. The resulting structure confirmed a highly similar arrangement to the human complex (Fig. 4D, Fig. S17). Despite this structural similarity, one notable difference is that the DI19 co-factor lacks the ZZ domain. Instead, an internal ZZ domain is found in AtUBR4 itself (Fig. S18). To explore whether the plant ZZ domain displays distinct specificity to HsKCMF1-ZZ we determined the co-crystal structure of the AtUBR4-ZZ in complex with an ^N^RS containing peptide (Fig. 4E, Fig. S13F, Table S3). We observed multiple differences in the substrate binding pocket, for example a specific interaction of the peptide’s R1 residue with a serine residue in the ZZ motif, whereas the corresponding residue in HsKCMF1 ZZ is a leucine. In agreement with this, ITC showed that the AtUBR4 ZZ domain binds to ^N^RA and ^N^RR peptide sequences (Fig. S13G), whereas HsKCMF1 ZZ domain did not. Thus, the AtUBR4 complex could act as a traditional type 1 N-degron receptor, consistent with *in vivo* studies (38).

## Discussion

Our data show that the evolutionarily conserved UBR4 complex forms a megadalton E4 arena, in which inward-facing ZZ-DZB domains of KCMF1 act not as E3 ligases but as substrate receptors. UBR4 recognizes pre-ubiquitinated substrates based on their orphan protein character (16), the presence of an MTS (17), or N-degrons (41). The pronounced preference for threonine in position-2 points to the targeting of processed mitochondrial proteins, eventually leaking from mitochondria into the cytosol, as can be now explored in vivo. The breadth of these partially degenerate signals accounts for the large number of reported UBR4 substrates.

Although the ZZ-DZB motif of KCMF1 is central for recognition, efficient capture likely depends on multivalent, low affinity interactions, supplied by accessory UBR4 domains such as the BP, UBR and BS2 that also line the inner cavity. Organism specific variations illustrate this principle: in plants, specificity depends on an internal AtUBR4 ZZ motif, whereas in nematodes two elongated helices at the N-terminus extend into the central cavity to capture substrates, presumably recognizing the hydrophobic surface of orphaned proteins. Beyond domain composition, the ring itself imposes an additional size filter that favors small, orphaned proteins over multi-protein complexes. The functional importance of the ring architecture is emphasized by its conserved shape and dimension in CeUBR4, despite pronounced differences in its helical scaffold and dimerization interfaces. Moreover, UBR4 disease mutations are spread throughout the ring scaffold, highlighting its crucial role for proper PQC function (Fig. S19A).

E4 ligases like UBR4 play a dual role in amplifying ubiquitination signals and refining specificity in protein degradation (42, 43). Rather than relying solely on the priming E3 ligase for substrate selection, our findings emphasize the equally crucial role of E4 ligases in enhancing specificity. Although both chain-initiating E3 ligases and chain-extending E4 ligases individually may exhibit weak substrate specificity, their sequential action yields an effective and specific proofreading mechanism to target aberrant proteins (Fig. S19B,C). Moreover, the likelihood of sequential recognition increases with a substrate’s residence time. For example, mitochondrial precursor proteins imported efficiently into mitochondria escape recognition, whereas precursors stalled in the cytosol under stress become marked for destruction. Such temporal control ensures that truly dysfunctional or defective proteins are removed but transient intermediates are spared. Sequential E3/E4 ligase action should also facilitate the formation of specialized PQC pathways towards distinct substrate types. HsUBR4/KCMF1/CALM1 operates alongside other broad PQC enzymes, such as HUWE1, BIRC6, UBR5, HERC1 and HERC2 (18, 44–48), each targeting distinct yet overlapping sets of substrates. This combinatorial setting allows for fine-tuned regulation of PQC pathways in specific cell types and physiological conditions. Through its intricate structure, selective targeting of pre-ubiquitinated substrates, and synergy with other PQC ligases, UBR4 is thus able to adopt a crucial role in cellular homeostasis, enabling the precise and efficient elimination of defective proteins.

## Supplementary Material

Supplementary Materials

## Data Availability

Cryo-EM maps and atomic coordinates have been deposited in the Protein Data Bank (PDB) with accession codes pdb_00009QWS, pdb_00009QWX, pdb_00009QWZ, pdb_00009QWU, pdb_00009QX0, pdb_00009QX1, pdb_00009QX2, pdb_00009QX5 and pdb_00009QT9 and the Electron Microscopy Data Bank (EMDB) with accession codes EMD-52491, EMD-53426, EMD-53430, EMD-53431, EMD-52494, EMD-53428, EMD-53432, EMD-52488, EMD-53425, EMD-52504, EMD-53433, EMD-53434, EMD-53435, EMD-52513, EMD-52516 and EMD-53348. Crystal structures have been deposited in the PDB with accession codes pdb_00009LGS, pdb_00009JNI and pdb_00009UPZ. Uncropped gels are provided in Fig. S21. The mass spectrometry proteomics data have been deposited to the ProteomeXchange Consortium via the PRIDE partner repository with the dataset identifier PXD063485.
