# Supplementary material for: Architecture of the UBR4 complex, a giant E4 ligase central to eukaryotic protein quality control
Source: Science. Author manuscript; Available in PMC 2025 Sep 27. (PMC7618180; doi:10.1126/science.adv9309)
Supplement: Supplementary Materials [file EMS209076-supplement-Supplementary_Materials.pdf]

Science
AAAS

Supplementary Materials for

Architecture of the UBR4 complex, a giant E4 ligase central to eukaryotic protein quality control

Daniel B Grabarczyk*[1], Julian F Ehrmann[1,2,3,4], Paul Murphy[1], Woo Seok Yang[5], Robert Kurzbauer[1], Lillie E Bell[1,2], Luiza Deszcz[1], Jana Neuhold[6], Alexander Schleiffer[1], Alexandra Shulkina[2,7,8], Juyeon Lee[5], Jin Seok Shin[5], Anton Meinhart[1], Gijs A Versteeg[7,8], Eszter Zavodszky[9], Hyun Kyu Song[5], Ramanujan S Hegde[9], Tim Clausen*[1,10]

Corresponding authors: DBG: Daniel.grabarczyk@imp.ac.at, TC: tim.clausen@imp.ac.at

The PDF file includes:

Materials and Methods
Figs. S1 to S21
Tables S1 to S5
References

Materials and Methods

Cloning and expression

UBR4, KCMF1 and CALM1 genes from *H. sapiens*, *C. elegans* and *A. thaliana* were codon-optimized for insect cell expression and, where required, synthesized in fragments flanked with BsaI sites. UBR4 was synthesized with a C-terminal His_{10} tag, KCMF1 with a C-terminal StrepII tag and CALM1 with an N-terminal FLAG tag. The three genes were assembled via scarless GoldenGate reactions into a pGBdest vector with each gene flanked by a polyhedrin promoter and an SV40 polyA signal. For monomeric KCMF1, the same procedure was used without UBR4 and CALM1. For monomeric HsUBR4 and CeUBR4 the same procedure was used except the His_{10} tag was replaced by a StrepII tag. For the AtUBR4 complex, DI19 and CALM1 were in a separate vector to AtUBR4 and were expressed together by co-infection. Mutations were generated by Gibson assembly. The HsUBR4/KCMF1/CALM1 plasmids was amplified in three equally sized fragments with complementary overhangs. Mutations were introduced in one fragment using a blunt-end ligation strategy. Specific domain deletions were ΔZZ-DZB (KCMF1, 2-132), ΔUBR (UBR4, 1651-1730), ΔBS2 (UBR4, 3543-3719) and ΔRING (UBR4, 4807-5183) The sequence of all generated insect cell constructs was confirmed by whole plasmid sequencing. Plasmids were transformed into DH10EmBacY cells and blue-white screening was used to select colonies with bacmids. Bacmids were extracted by alkaline lysis and isopropanol precipitation and then transfected using Polyethylenimine PEI 25K into Sf9 cells (Expression Systems) for viral amplification. Protein expression was performed in *Trichoplusia ni* High-Five cells (Accession: CVCL_C190) (Thermo Fisher). Cells were infected with virus at a density of 1.5 x 10^6 ml^{-1} and grown for three days at 27°C. Cells were harvested by centrifugation at 600 x g.

Constructs for *E. coli* expression were generated by Gibson assembly. His_6-SUMO-MTS1/2-linker-UbΔGG was synthesized with overhangs and inserted into a pET28 vector. The linker comprised 21 glycine or serine residues. The control construct with no MTS as well as N-degron versions were generated by blunt-end mutagenesis targeting the region directly after the SUMO cleavage site and before the linker. All substrate plasmids were generated by synthesis of the TnSubstrate genes followed by Gibson assembly into the pET28 His_6-SUMO-MTS1/2-linker-UbΔGG where substrate replaced either the MTS for substrate-Ub* constructs or replaced the MTS-linkerUb-ΔGG for substrates without fused ubiquitin. Plasmids were transformed into BL21 (DE3) cells (Thermo Fisher: EC0114) for expression in Lysogeny Broth media. Expression was performed at 17°C with 0.2 mM isopropyl-ß-d-thiogalactopyranoside.

The DNAs for the ZZ-domain of KCMF1^{ZZ+DZB} (residues 2~142) and BIG^{ZZ} (residues 2612~2669) were synthesized with optimized codon usage (IDT, Integrated DNA Technologies). These DNAs were inserted into the His-LC3B vector for using LC3B-fusion technique (*49*), which is originated from pET expression vector (Novagen). Later, the linker (residues 55~76) between the ZZ and DZB domain of KCMF1^{ZZ+DZB} construct was replaced with Gly x 4 by mutagenesis (KCMF1$^{ZZ+DZB(\triangle linker)}$) for better crystallization. The N-degron sequence of BIG^{ZZ} (R-BIG^{ZZ}) was also introduced into the plasmid for the N-degron complex crystallization. These proteins were expressed in *E.coli* BL21(DE3) induced by adding 0.5 mM isopropyl β-D-1-thiogalactopyranoside, and 400 µM of $ZnSO_4$ was also added to the culture medium.

Protein purification

HsUBR4/KCMF1/CALM1 and AtUBR4 expression pellets were resuspended in buffer containing 50 mM HEPES pH 7.5, 500 mM NaCl, 0.5 mM TCEP, 20 mM imidazole with

benzonase (MBS) and a Complete EDTA-free protease inhibitor tablet (Roche). Cells were lysed using a glass douncer and cleared by centrifugation at 40,000 x g. The soluble lysate was loaded on a 5 mL HisTrap HP (Cytiva) column using an Akta PURE system (Cytiva). The column was washed with 7 column volumes (CVs) of the lysis buffer followed by 6 CVs of buffer with 80 mM imidazole. UBR4 complexes were then eluted with a linear gradient from 80 to 300 mM imidazole. SDS-PAGE was used to identify fractions containing UBR4. For the HsUBR4/KCMF1/CALM1 co-expression construct, the eluted protein was concentrated by ultrafiltration to 2 mL and subjected to size-exclusion chromatography using a Superose 6 pg 16/70 column (Cytiva) equilibrated in 20 mM HEPES pH 7.5, 250 mM NaCl, 0.5 mM TCEP. Fractions containing HsUBR4/KCMF1/CALM1 were concentrated by ultrafiltration, flash frozen and stored at -70°C. AtUBR4 was purified by anion-exchange chromatography using a 6 mL Resource Q column (Cytiva) instead of SEC after the His-affinity purification in 50 mM HEPES pH 7.5, 0.5 mM TCEP and a 150-1000 mM NaCl gradient. Fractions containing AtUBR4 were pooled, concentrated and flash frozen.

For the CeUBR4/KCMF1 complex, lysis followed the same protocol except imidazole was excluded from the buffer. The clarified lysate was first purified by Strep-affinity chromatography using a 5 mL StrepTrap HP column (Cytiva) equilibrated in lysis buffer. The column was washed with 8 CVs of the same buffer and then eluted in the same buffer with 2.5 mM desthiobiotin. The complex was subjected to SEC using the same protocol as for HsUBR4/KCMF1/CALM1. Following SEC, the complex was loaded on a 1 mL HisTrap HP column, washed with 20 mM and then 50 mM imidazole and eluted with 300 mM imidazole. The imidazole was then removed by repeated concentration and dilution using a spin concentrator and flash frozen and stored at -70°C.

For HsUBR4 and CeUBR4 monomeric complexes, cell pellets were resuspended in phosphate buffered saline with 0.5 mM TCEP (PBS-TCEP) and lysed and subjected to Strep-affinity chromatography as above. The proteins were further purified by anion-exchange chromatography using a 6 mL Resource Q column (Cytiva) in PBS-TCEP with and a 250-500 mM NaCl gradient. Fractions containing UBR4 were pooled, concentrated and flash frozen.

Expression pellets for the SUMO-linker-Ub* constructs were resuspended in 50 mM Tris-HCl pH 8.0, 500 mM NaCl, 0.5 mM TCEP, 25 mM imidazole, benzonase and protease inhibitors and lysed by sonication. Clarified lysate was applied to a 5 mL HisTrap HP (Cytiva) using a syringe, washed with 5 CVs of the same buffer and then eluted with 250 mM imidazole in the same buffer. The eluate was treated with his_6-SENP2 protease (MBS) overnight at 4oC to remove the SUMO and expose the N-degron. The imidazole concentration was reduced to 25 mM imidazole by dilution in 50 mM Tris-HCl pH 8.0, 500 mM NaCl, 0.5 mM TCEP and the sample was reapplied through the 5 mL HisTrap HP column using a syringe to remove his_6-SUMO and his_6-SENP2. The column was washed with an additional 3 CVs of buffer with 25 mM imidazole and all flow-through collected, concentrated and then flash frozen and stored at -70°C. For SUMO-TnSubstrate constructs the same procedure was used except the buffers contained 400 mM ammonium sulfate instead of 500 mM NaCl.

The cells expressing KCMF1$^{ZZ+DZB(\triangle linker)}$ and R-BIG^{ZZ} were lysed by sonication with lysis buffer (50 mM Tris-HCl pH 8.0, 200 mM NaCl, and 0.5 mM TCEP (tris(2-carboxyethyl)phosphine)). Target proteins were purified by affinity chromatography (HisTrap^{TM} HP, Cytiva, 17524802), His-LC3B tag cleavage by ATG4B protease (20 ^{o}C, 12 hr) (*50*), anion exchange chromatography (HiTrap^{TM} Q HP, Cytiva, 17115301), and size-exclusion

chromatography (HiLoad™ Superdex™ 75 16/600 pg) with final buffer (25 mM Tris-HCl pH 7.5, 150 mM NaCl, 0.5 mM TCEP).

UBE2D3, UBE2A, UBA1, and ubiquitin were purified as previously described (*28*, *46*). The concentration of all proteins was determined by absorbance at 280 nm using calculated extinction coefficients.

Crystallography

The purified KCMF1$^{ZZ+DZB(\triangle linker)}$ and R-degron-fused BIG^{ZZ} were concentrated to 15~20 mg/ml. The RC$_{O3}$ and RT peptides were mixed with KCMF1$^{ZZ+DZB(\triangle linker)}$ with 3:1 ratio (peptide:KCMF1$^{ZZ+DZB(\triangle linker)}$). Crystallization was performed at 20 ^{o}C using the sitting-drop vapor diffusion method, mixed in a ratio of 1:1 with crystallization solutions using a Gryphon machine (Art Robbins Instruments). Initial crystals were generated within the crystallization solutions as follows; KCMF1$^{ZZ+DZB(\triangle linker)}$ with RC$_{SO3}$ peptide – 30% (v/v) Precipitant Mix 1, 0.1 M Buffer System 3 pH 8.5, and 0.06 M Divalents [Morepheus, A9 condition: Molecular Dimensions, MD1-46], KCMF1$^{ZZ+DZB(\triangle linker)}$ with RT peptide – 37.5 % (v/v) Precipitant Mix 4, 0.1 M Buffer System 2 pH 7.5, 0.06 M Divalents [Morepheus, A8 condition: Molecular Dimensions, MD1-46] R-BIG^{ZZ} – 15% (w/v) polyethylene glycol 3,350, 0.1 M HEPES sodium pH 7.0, 0.01 M magnesium chloride hexahydrate, and 5.0 mM nickel(II) chloride hexahydrate [PEG/ION, H7 condition: Hampton Research, HR2-139]. R-BIG^{ZZ} crystals were reproduced within the solution containing 7.5% (w/v) polyethylene glycol 3,350, 0.1 M HEPES sodium pH 7.0, 0.01 M magnesium chloride hexahydrate, and 5.0 mM nickel(II) chloride hexahydrate in addition to 10% (v/v) 0.1 M strontium chloride hexahydrate (Additive Screen, A8 condition: Hampton Research, HR2-138). 30~40% (v/v) glycerol solution was mixed with mother liquors as cryo-protectants. The crystals were flash-frozen in liquid nitrogen. All the X-ray data was collected at Pohang Accelerator Laboratory (PAL) BL 5C in South Korea. Phasing and refinement were carried out with the Phenix software package (*51*). The phases of crystal data were determined using molecular replacement (MR) with the AlphaFold and previous experimental models. These models were rebuilt manually and refined in iterative cycles with Coot (*52*). Final structural models were validated with Molprobity

Cryo-EM grid preparation and data collection

1.3 mg/ml HsUBR4/KCMF1/CALM1 or CeUBR4/KCMF1 in 25 mM HEPES pH 7.5, 200 mM NaCl, 0.5 mM TCEP was applied onto a freshly glow-discharged (90 seconds at 25 mA) Quantifoil R1.2/1.3 Cu 200 mesh grid. Grids were blotted for 1.2 seconds before rapid freezing in liquid ethane using a Leica GP2 plunge-freezer. For the HsUBR4/KCMF1/CALM1/UBE2A complex the protein was first incubated with 3 μM UBE2A (a 1.5 fold excess). For EGTA treatment the sample was first incubated with 2 mM EGTA for 30 minutes. For all cases except one HsUBR4/KCMF1/CALM1 dataset, 0.8 mM CHAPSO was added to the sample directly before freezing. A Glacios TEM (Thermo Fisher) equipped with a Falcon 3 detector was used to screen grids. For data collection, the grids were transferred to a Titan Krios G4 (Thermo Fisher) with a Falcon 4EC detector operated by the Research Institute of Molecular Pathology, Austria. All data were collected with the same parameters using the EPU software (Thermo Fisher) with 130,000x magnification (0.951 Å pixel size) and a total dose of 50 e/Å^2. Patch Motion correction was performed on-the-fly using CryoSPARC Live (*53*).

Cryo-EM data analysis

The HsUBR4/KCMF1/CALM1 structure was generated by combining three different datasets – one without CHAPSO, one with CHAPSO and one with CHAPSO and EGTA. The HsUBR4/KCMF1/CALM1/UBE2A and CeUBR/KCMF1 structures were from single datasets. All datasets were processed using the same strategy with specifics shown in Figs S2, S6, S8 and S13. Motion corrected micrographs were simultaneously imported into CryoSPARC v4 (*53*) and Relion 4.0 (*54*). CTF correction, particle picking and removal of junk particles was performed independently with both programs. In CryoSPARC, patch CTF estimation followed by blob picking was used to generate templates for template picking. After template picking, particles were extracted in a 512 pixel box size downscaled to 128 pixels. Many rounds of 2D classification were performed until only particles with clear secondary structure remained. For 2D classification, 100 classes were used with a batch size of 200 and 60 iterations. All initial models were generated by Ab Initio in CryoSPARC. In Relion 4, after CTF estimation with CTFFIND4, 1000 particles were manually picked to generate templates for autopicking. Autopicked particles were extracted at 512 pixels and downscaled to 100 pixels. Repeated round of 2D classification were used to only remove ice and edges. 2D classification was performed with 100 classes using a 360 Å mask using the EM algorithm. 3D classification with alignment using a 360 Å mask with the CryoSPARC initial model lowpass filtered to 30 Å was then used to remove low quality particles.

These two particle sets were then combined in CryoSPARC, duplicates were removed by performing 2D classification and removing particles within 80 Å of each other. Particles were then extracted with a box size of 512 pixels, downscaled to 384 pixels (1.27 Å/px). The extracted particle stack was then imported into Relion for all further processing. To obtain clear density for the co-purified substrate in the CeUBR4/KCMF1 structure a 3D classification with alignment was performed. For all other maps, first a 3D refinement was performed with either C1 or C2 symmetry enforced using a global mask and the upscaled map from the particle cleaning 3D classification lowpass filtered to 30 Å. The particles refined with C2 symmetry were subjected to symmetry expansion. Then soft masks were generated for the various parts of the structure and 3D classification without alignment was used using either the C1, C2 or symmetry expanded aligned particles and different T values (detailed in the relevant processing figure) to find the best particle sets for each region of the structure. These particles were subjected to either global or focused refinement to obtain the final maps. A composite map was made for main figures and model to map fit figures in ChimeraX by aligning the focused maps onto a single map which had all features moderately well resolved. Certain regions were selected based on the molecular model, scaled and combined with the volume maximum command.

The AtUBR4 complex was entirely processed in CryoSPARC. Initial processing followed the same protocol as for other datasets, except blob picking and Topaz picking were additionally used to increase the number of particles. Topaz was trained on 20 micrographs with default parameters and then used to pick the entire dataset. After 2D classification, combination of the three particle sets and removal of duplicates, particles were extracted with a box size of 512 pixels, downscaled to 384 pixels (1.27 Å/px). A homogeneous refinement was first performed with C2 symmetry enforced, a static global mask and an initial model lowpass filtered to 30 Å. Due to very poor density outside of the C-term dimerization core, we only pursued local refinement of the well-resolving region. For this, a soft mask was generated for all regions where there was clear secondary structure and this was used along with the outputs from the homogeneous refinement for a local refinement with default parameters. 3D FSC curves for all Relion-refined structures are shown in **Fig. S20**.

Molecular model building and refinement

The C-terminal and N-terminal dimerization regions of the HsUBR4/KCMF1/CALM1 structure were autobuilt using ModelAngelo (*55*). For the CeUBR4/KCMF1 structure, ModelAngelo was used to build the C-terminal dimerization region as well as the region comprising the BP domain and associated ARM repeats. From these starting models we used rounds of modelling in Coot (*52*) and real space refinement in Phenix Real Space Refine (*51*) to obtain models which fit well to the density while having reasonable geometry statistics. Model to map fits for these regions are shown in **Fig. S3** and **S15** while refinement statistics are shown in **Table S1** and **Table S4**).Additionally, many AlphaFold3 models were predicted for all sections of the structure including the various protein-protein and protein-zinc interactions using the AlphaFold3 server (*24*). These were generally accurate enough at a local level to be docked directly and confidently in the map (**Fig. S4, Fig. S16, Fig. S17**). However, they differed significantly from the real structure at a global conformational level. When secondary structure was clear in these maps, rigid body fitting in Coot (*52*) was used to correct differences in long-range conformation and sidechains were deleted before real space refinement in Phenix Real Space Refine (*51*). Refinement statistics are shown in **Table S1** and **Table S4**. For maps without clear secondary structure features, the AlphaFold3 model was directly docked and used for figures and no further modelling or PDB deposition was done.

Ubiquitination assays

All ubiquitination assays were performed in buffer containing 25 mM HEPES pH 7.5, 150 mM NaCl, 0.5 mM TCEP, 5 mM $MgCl_2$. Specific details of reaction conditions are provided in the respective figure legends. Unless otherwise indicated, reactions contained 100 or 200 nM UBR4 monomer as indicated, 250 nM UBA1, 500 nM UBE2A, 5 μM substrate-Ub*, 2.5 μM UBE2D3 when present, and either 5 μM Ub-K0 with or without 0.5 μM DyLight488-Ub-K0 as indicated, or 10 uM wild-type ubiquitin spiked with 1 μM Ub-DyLight488. Reactions were initiated by the addition of 5 mM ATP and proceeded for the time indicated in the figure legend at 37°C. Reactions were quenched with SDS gel loading buffer and separated by SDS-PAGE using 4-12% NuPAGE Bis-Tris gradient gels (Invitrogen) in MES running buffer. If required, gels were first imaged for DyLight488 fluorescence using a ChemiDoc MP system (Bio-Rad) before Coomassie staining. For the Stain-Free gel, a Bio-Rad Mini-PROTEAN TGX 4-15% gel was run in tris-glycine buffer and imaged using the ChemiDoc MP system (Bio-Rad). All experiments were technically replicated at least twice while quantified experiments were performed in triplicate. For quantification the intensity of the product band was measured in ImageJ and presented as a ratio to the product in the control condition after background subtraction

Mass photometry

Experiments were performed using a OneMP mass photometer (Refeyn Ltd.) controlled by the AcquireMP application. A one-minute acquisition time in the medium field of view was used. To obtain average species masses, particle event histograms were automatically Gaussian fitted with the DiscoverMP software. Samples were first prepared in a 200 nM stock solution in 25 mM HEPES pH 7.5, 150 mM NaCl, 0.5 mM TCEP and prior to measurement were diluted 10x in PBS. Data were plotted in the DiscoverMP application.

Isothermal titration calorimetry

Purified GST- BIG^{ZZ} and GST- KCMF1^{ZZ+DZB} were prepared at a final concentration of 50 μM in ITC buffer (50 mM Tris-HCl at pH 8.0, 150 mM NaCl, and 1 mM TCEP) and Arg/N-degron peptides (R-R, R-L, R-D, R-T, R-CO3) were dissolved in the same buffers at a concentration of 0.5 mM. Measurements were carried out at 25 °C using a Microcal PEAQ-ITC (Malvern). A total of 19 injections (2μl per injection) were made into 280μl samples of each protein. The experimental data was calculated using the embedded analyzing software package provided with the instrument. At least two experiments were performed under varying concentration conditions.

Circular dichroism spectroscopy

Samples for circular dichroism spectroscopy were prepared in 150 mM sodium phosphate pH 7.5 at 0.2 mg/ml. Circular dichroism spectra were collected using a Chirascan Plus (Applied Photophysics Ltd.) spectrometer that enables simultaneous measurement of CD and absorbance spectra. CD signals were recorded over a 180-280 nm range, using a 0.5 mm cell. CD spectra of protein samples or the buffer were scanned with a 0.5 s integration, 0.5 nm step resolution, and 1 nm bandwidth. Each spectrum is the average of 3 scans; thus, the total integration time for each point of the spectrum is 1.5 s. Prior to spectra measurements, the CD baseline was recorded with an empty cell holder and with another 1.5 s integration. From each recorded spectrum of protein samples, the corresponding smoothed buffer spectrum was subtracted. All spectroscopic measurements were carried out at 20 ± 0.01 °C.

Mass spectrometry

Samples were treated with trypsin, Lys-C or Asp-N protease in ammonium bicarbonate before extraction with 5% formic acid. Peptide desalting was performed using an Oasis HLB 96-well µElution Plate with 2 mg Sorbent (Waters) and organic content of the eluates were removed by evaporation in a vacuum centrifuge.

The nano HPLC system used was an UltiMate 3000 RSLC nano system (Thermo Fisher Scientific) coupled to a Q Exactive HF-X mass spectrometer (Thermo Fisher Scientific), equipped with a Proxeon nanospray source (Thermo Fisher Scientific). Peptides were loaded onto a trap column (Thermo Fisher Scientific, PepMap C18, 5 mm × 300 μm ID, 5 μm particles, 100 Å pore size) at a flow rate of 25 μL min^{-1} using 0.1% TFA as mobile phase. The trap column was switched in line with the analytical column (Thermo Fisher Scientific, PepMap C18, 500 mm × 75 μm ID, 2 μm, 100 Å) after 10 minutes and peptides were then eluted using a binary one hour gradient, starting from 98% A (water/formic acid, 99.9/0.1, v/v) and 2% B (water/acetonitrile/formic acid, 19.92/80/0.08, v/v/v), increasing to 35% B over 60 minutes and then to 95% B over 5 minutes with a flow rate of 230 nl min^{-1}.

The Orbitrap Exploris 480 mass spectrometer was operated in data-dependent mode, performing a full scan (m/z range 350-1200, resolution 60,000, normalized AGC target 100%) at 3 different compensation voltages (CV -45, -60, -75), followed each by MS/MS scans of the most abundant ions for a cycle time of 1 second per CV. MS/MS spectra were acquired using HCD collision energy of 30, isolation width of 1.2 m/z, orbitrap resolution of 30,000, normalized AGC target 200%, minimum intensity of 50,000 and maximum injection time of 100 ms. Precursor ions selected for fragmentation (include charge state 2-6) were excluded for 20 s. The monoisotopic precursor selection (MIPS) filter and exclude isotopes feature were enabled.

For peptide identification, the RAW-files were first loaded into Proteome Discoverer (version 2.5.0.400, Thermo Scientific). MS/MS spectra were then searched using MSAmanda

v2.0.0.19924. The fragment and peptide mass tolerance were set to ±10 ppm and the maximum number of missed cleavages was set to 2, using semi-tryptic enzymatic specificity without proline restriction. Peptide and protein identification was initially performed by searching the RAW-files against the Uniprot-database using the taxonomy Trichoplusia ni (2021-03; 21,163 sequences; 13,598,141 residues) supplemented with common contaminants and the sequences of UBR4, KCMF1 and CALM1 using Beta-methylthiolation on cysteine as a fixed modification. The result was filtered to 1 % FDR on protein level using the Percolator algorithm integrated in Proteome Discoverer. Following this, a second round of searching was done on the sub-databases of these proteins looking for protein modifications and again filtered. Peptides were subjected to label-free quantification using IMP-apQuant. Proteins were filtered to be identified by a minimum of 2 PSMs in at least 1 sample. Identified proteins were pre-filtered to contain at least 3 quantified peptide groups.

Phylogenetic analysis

Ubr4 and KCMF1/DI9 sequence families were collected with NCBI blast searches (BLASTP 2.8.1+ in NCBI non redundant protein or UniProt reference proteomes databases (Uniprot 2023), using Homo sapiens Ubr4 (UniProt: Q5T4S7), Homo sapiens Kcmf1 (Q9P0J7) or Arabidopsis thaliana DI19-1 (Q39083) as reference. Sequences were selected for a high domain coverage and a wide taxonomic range, and aligned with mafft (v7.525, -linsi method). For tree inference, the respective domains were extracted with Jalview and columns covering less than 40% of the sequences were removed. A maximum likelihood phylogenetic tree was calculated with IQ-TREE 2 (v.2.2.0,) with standard model selection using ModelFinder. The tree was visualized in iTOL (v6). The branch length represents the expected number of substitutions per site.

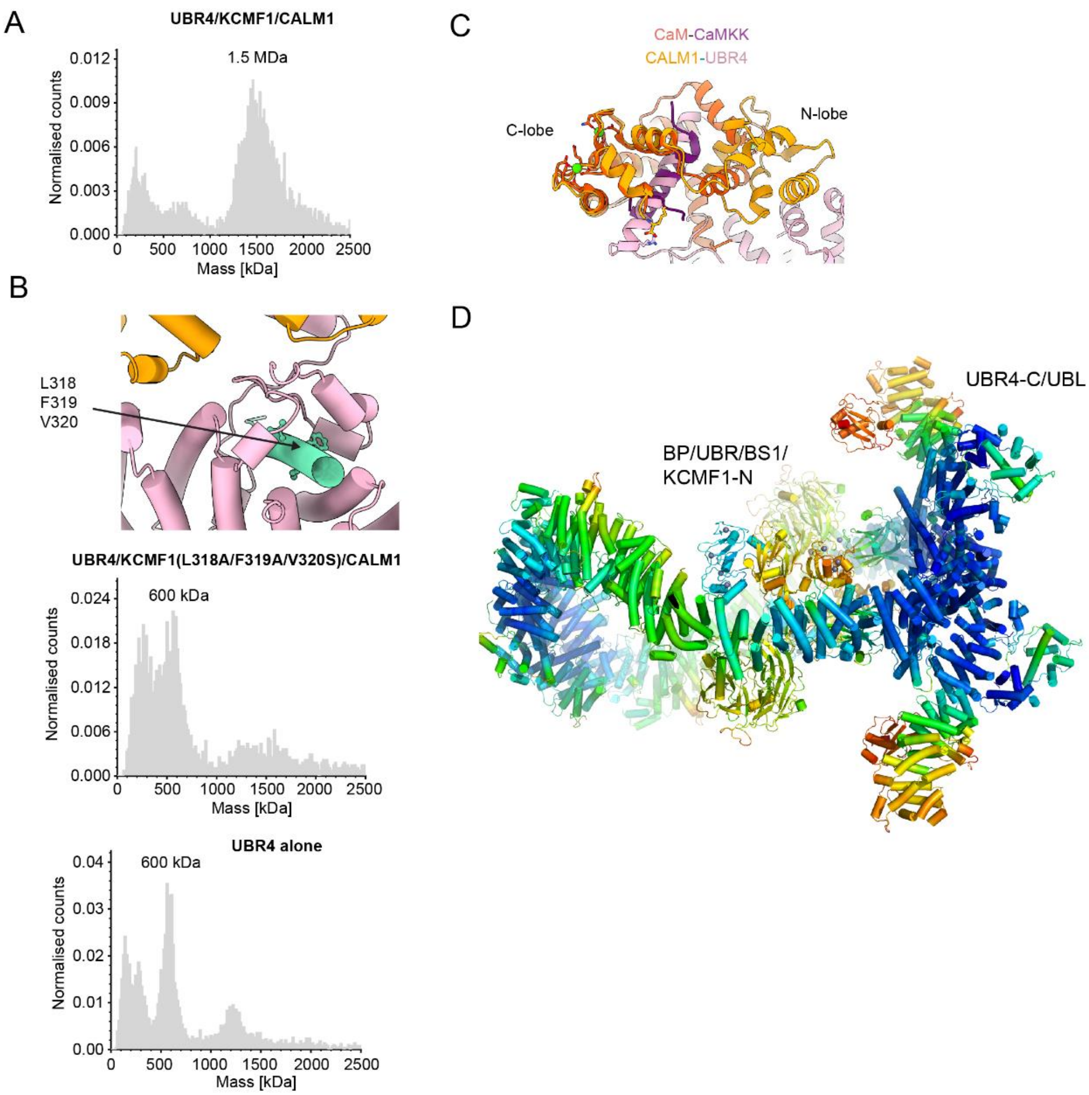

Fig. S1 – Assembly and structure of the HsUBR4/KCMF1/CALM1 complex. (A-C) Mass photometry histograms to determine the molecular weight distribution of **(A)** the co-expressed complex, **(B)** the UBR4/KCMF1/(L318A/F319A/V320S)/CALM1complex with UBR4 expressed alone as a reference. **(C)** Comparison of the HsUBR4-HsCALM1 interaction with a canonical CALM interaction (calmodulin-calmodulin dependent protein kinase kinase, pdb_00001IQ5). Calcium ions are represented as green spheres. **(D)** Cryo-EM model of the HsUBR4/KCMF1/CALM1 complex refined in the global map colored by *B* factor to indicate the flexibility of different regions. Red indicates a higher *B* factor and blue lower.

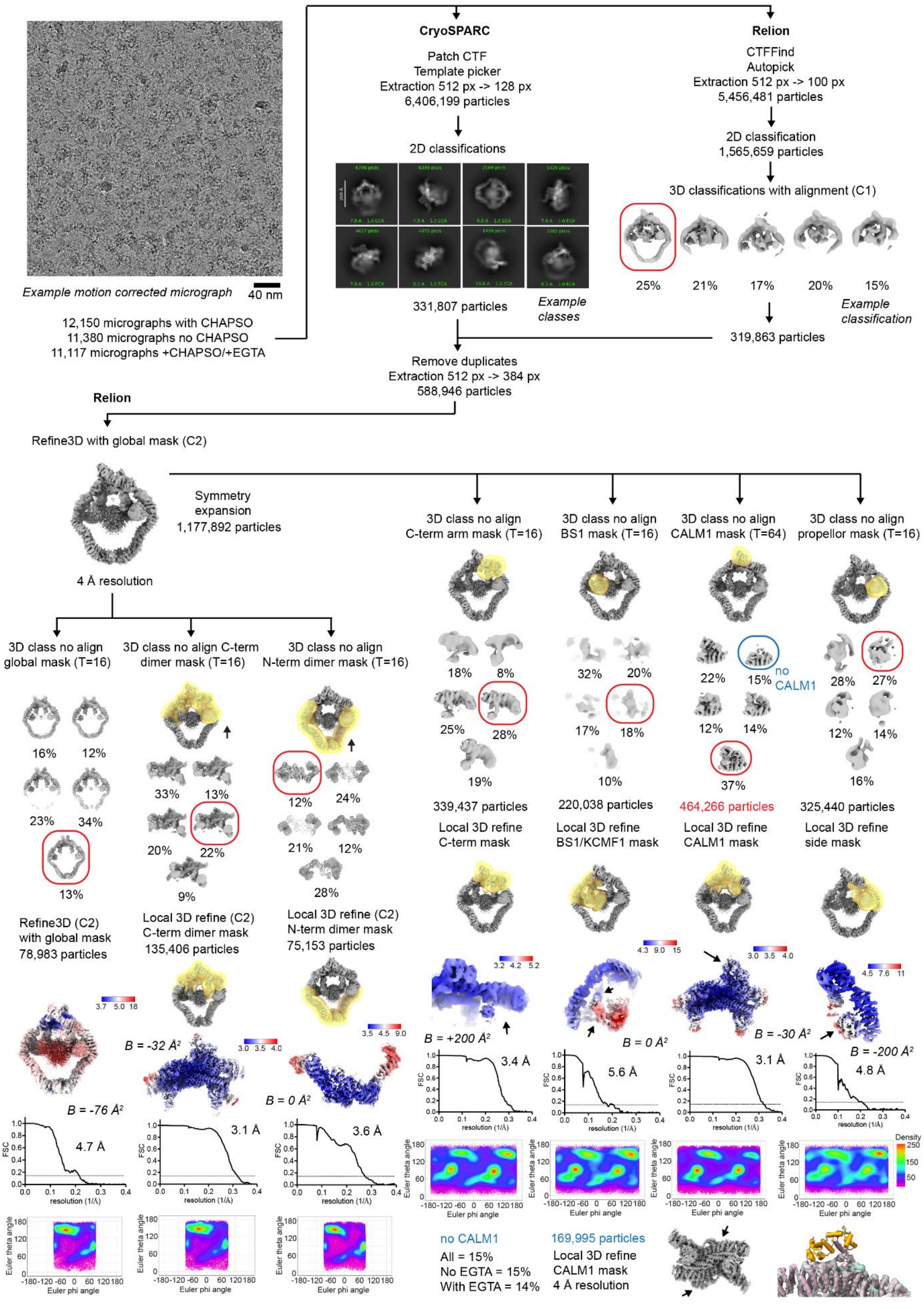

Example motion corrected micrograph
40 nm
12,150 micrographs with CHAPSO
11,380 micrographs no CHAPSO
11,117 micrographs +CHAPSO/+EGTA
CryoSPARC
Patch CTF
Template picker
Extraction 512 px -> 128 px
6,406,199 particles
2D classifications
331,807 particles
Example classes
Relion
CTFFind
Autopick
Extraction 512 px -> 100 px
5,456,481 particles
2D classification
1,565,659 particles
3D classifications with alignment (C1)
25% 21% 17% 20% 15%
Example classification
319,863 particles
Remove duplicates
Extraction 512 px -> 384 px
588,946 particles
Relion
Refine3D with global mask (C2)
Symmetry expansion
1,177,892 particles
4 Å resolution
3D class no align global mask (T=16)
16% 12% 23% 34% 13%
Refine3D (C2) with global mask
78,983 particles
B = -76 Å²
4.7 Å
3D class no align C-term dimer mask (T=16)
33% 13% 20% 22% 9%
Local 3D refine (C2) C-term dimer mask
135,406 particles
B = -32 Å²
3.1 Å
3D class no align N-term dimer mask (T=16)
12% 24% 21% 12% 28%
Local 3D refine (C2) N-term dimer mask
75,153 particles
B = 0 Å²
3.6 Å
3D class no align C-term arm mask (T=16)
18% 8% 25% 28% 19%
339,437 particles
Local 3D refine C-term mask
B = +200 Å²
3.4 Å
3D class no align BS1 mask (T=16)
32% 20% 17% 18% 10%
220,038 particles
Local 3D refine BS1/KCMF1 mask
B = 0 Å²
5.6 Å
3D class no align CALM1 mask (T=64)
22% 15% no CALM1 12% 14% 37%
464,266 particles
Local 3D refine CALM1 mask
B = -30 Å²
3.1 Å
3D class no align propellor mask (T=16)
28% 27% 12% 14% 16%
325,440 particles
Local 3D refine side mask
B = -200 Å²
4.8 Å
FSC
resolution (1/Å)
Euler theta angle
Euler phi angle
Density
no CALM1
All = 15%
No EGTA = 15%
With EGTA = 14%
169,995 particles
Local 3D refine CALM1 mask
4 Å resolution

Fig. S2 – Cryo-EM processing pipeline for the HsUBR4/KCMF1/CALM1 complex. There is further analysis of CALM1 occupancy in the bottom right of the figure. 3D classification showed that CALM1 is substoichiometric but the percentage of particles without CALM1 on one side is the same in the datasets with or without EGTA. The model of the full complex is shown docked into the map missing one CALM1 molecule in the bottom right.

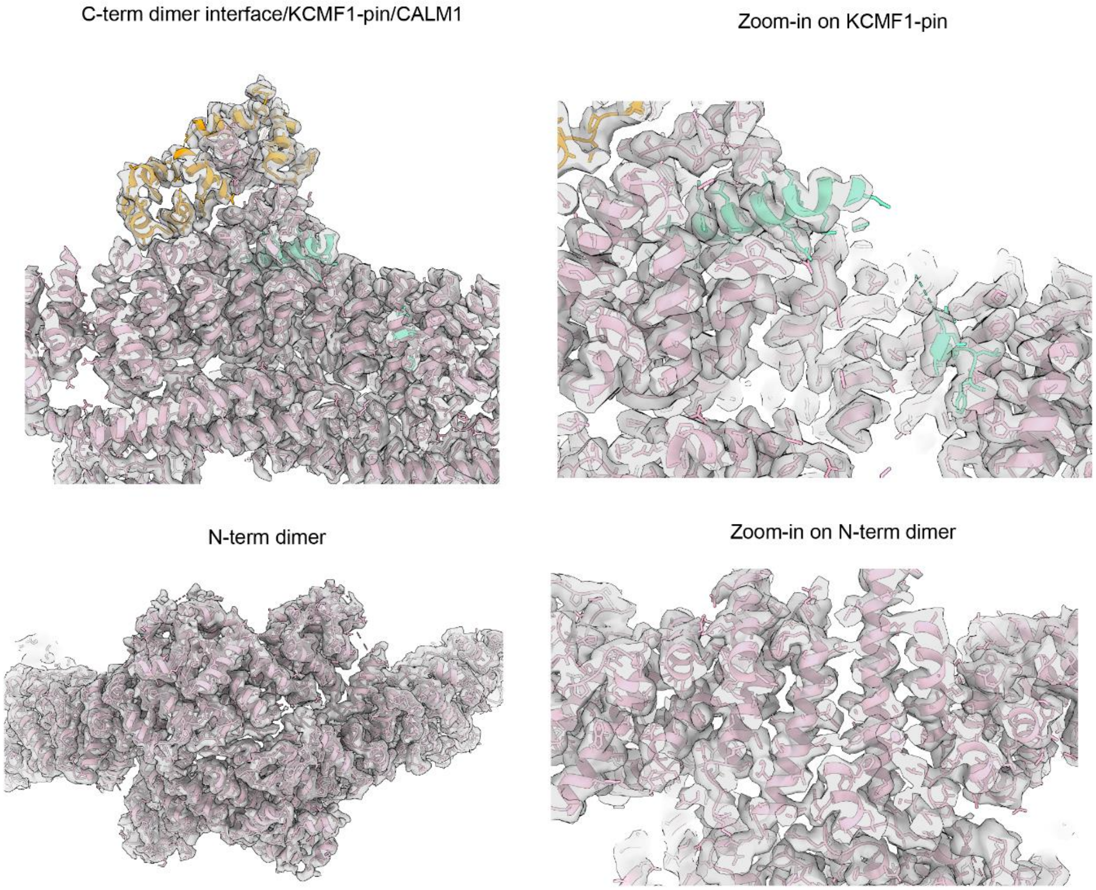

Fig. S3 – Example map to model fits for different regions of the HsUBR4 complex structure which were of sufficient resolution to build de novo using ModelAngelo.

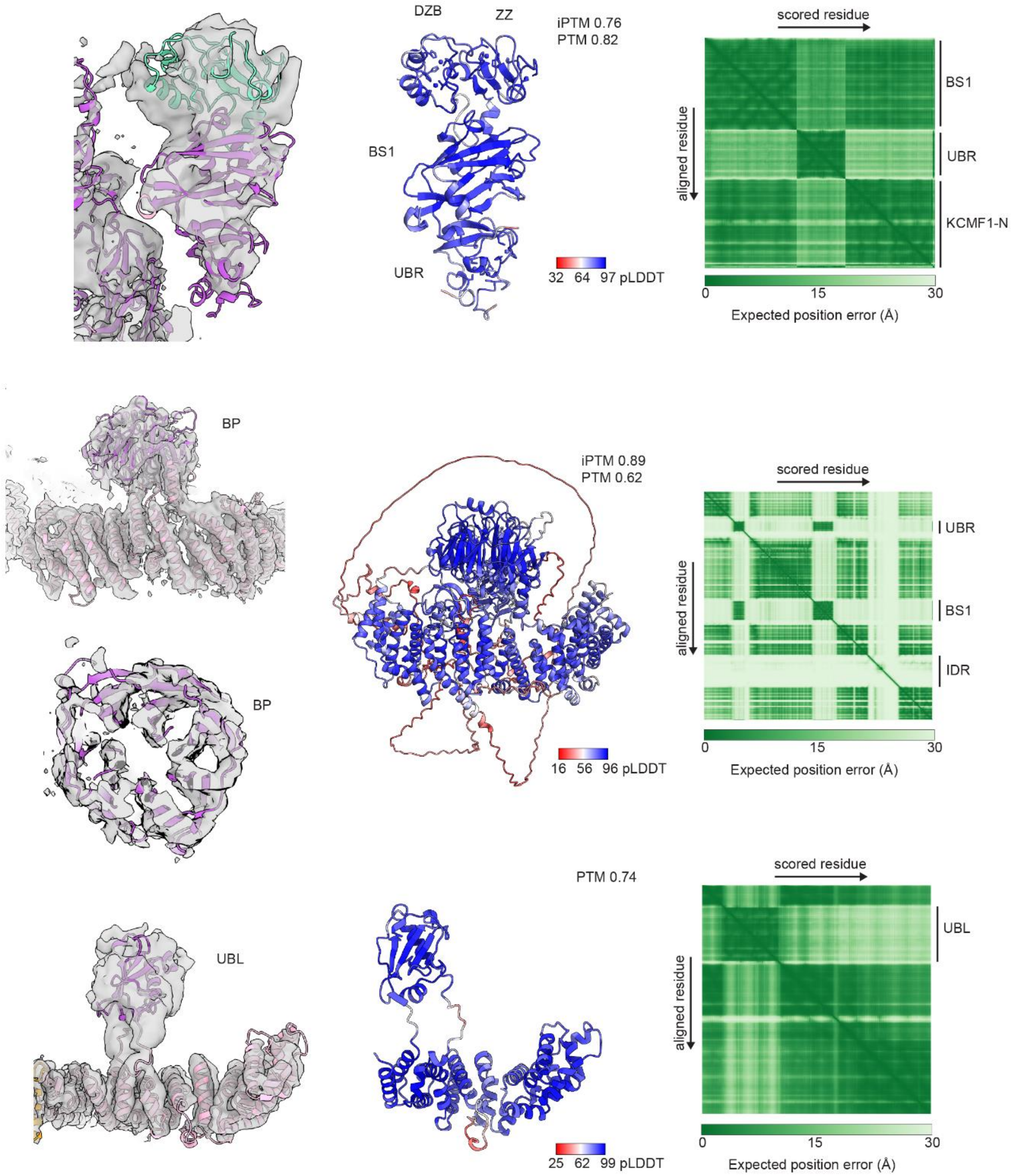

Fig. S4 – AlphaFold3 models for sections of the HsUBR4 complex with map resolutions too low for de novo modelling. The pLDDT, PAE, PTM and iPTM scores are shown as indicators of confidence for each AlphaFold3 prediction as well as the model to map fit of these models into the experimental cryo-EM density.

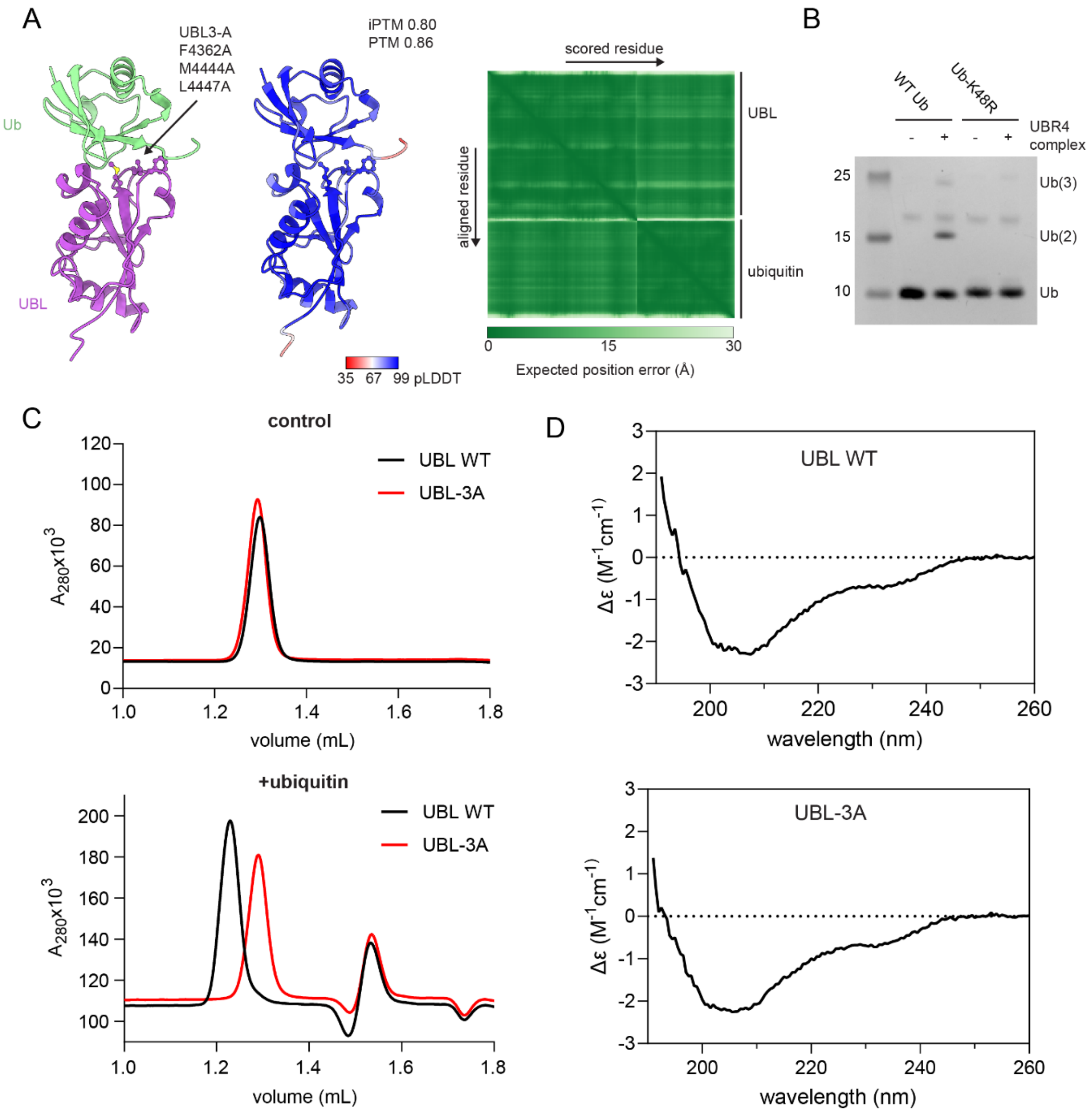

Fig. S5 – The UBL-Ub interaction. (A) AlphaFold3 model of the HsUBR4 UBL domain together with ubiquitin with the residues mutated in the UBL-3A variant indicated. pLDDT, PAE, PTM and iPTM scores are shown for the prediction. **(B)** Ubiquitination assay for 30 minutes at 37°C with 200 nM HsUBR4 complex, 250 nM UBA1, 500 nM UBE2A and 10 µM of either WT or K48R ubiquitin. **(C)** Analytical size-exclusion chromatography experiment to investigate binding of the WT UBL and UBL-3A variant with ubiquitin. In control runs, 200 µM UBL variant was injected on a Superose 6 column equilibrated in buffer containing 25 mM HEPES pH 7.5, 25 mM NaCl, 2 mM TCEP. In runs with ubiquitin, 200 µM UBL variant was pre-incubated with 600 µM ubiquitin and run on a Superose 6 column equilibrated in buffer containing 25 mM HEPES pH 7.5, 25 mM NaCl, 2 mM TCEP and 300 µM ubiquitin. Inclusion of ubiquitin in the buffer was necessary due to the highly transient nature of the interaction. **(D)** Circular dichroism spectra of the indicated purified HsUBR4 UBL domain variants.

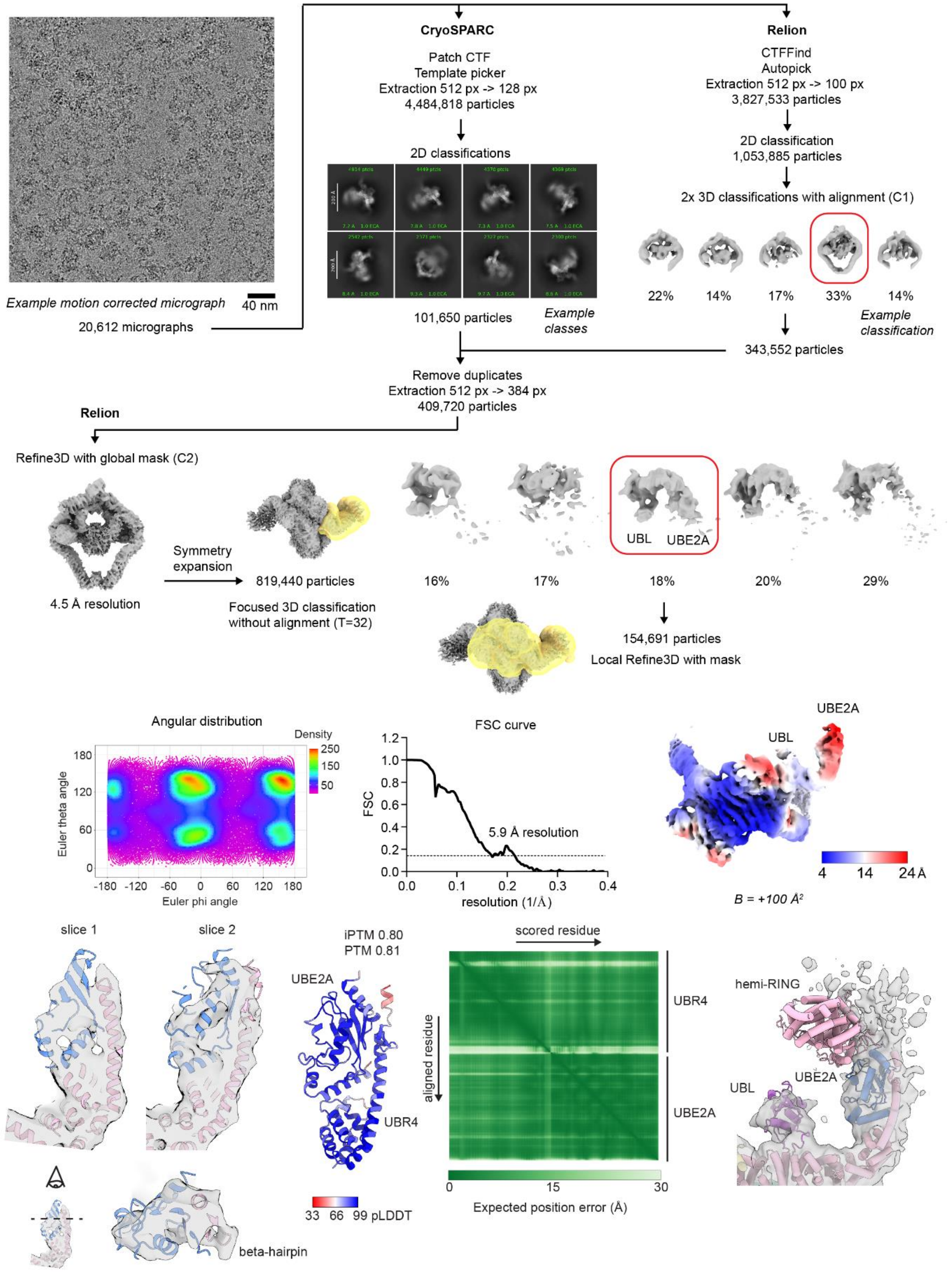

Fig. S6 – Cryo-EM processing pipeline for the HsUBR4/KCMF1/CALM1 complex incubated with HsUBE2A. At the bottom of the figure, details of the AlphaFold3 prediction to

model the complex are shown along with slices showing model to map fit. In the bottom right is an overlay of the structure with the crystal structure of the UBE2A/hemi-RING complex (pdb_00008BTL) showing that the diffuse hemi-RING density is not consistent with an engaged conformation of the UBE2A/hemi-RING complex.

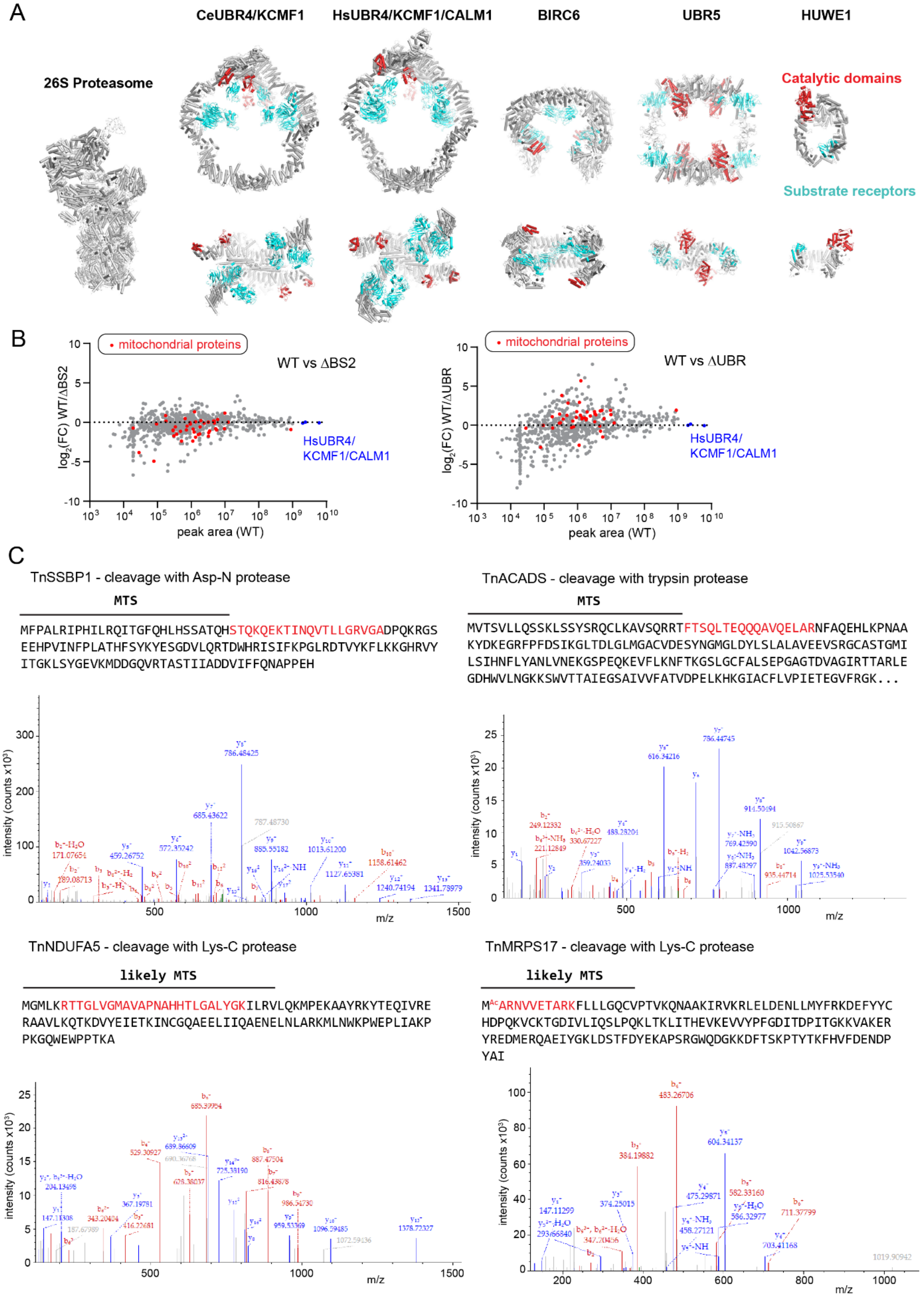
A
26S Proteasome
CeUBR4/KCMF1
HsUBR4/KCMF1/CALM1
BIRC6
UBR5
HUWE1
Catalytic domains
Substrate receptors
B
• mitochondrial proteins
WT vs ΔBS2
log2(FC) WT/ΔBS2
HsUBR4/
KCMF1/CALM1
peak area (WT)
• mitochondrial proteins
WT vs ΔUBR
log2(FC) WT/ΔUBR
HsUBR4/
KCMF1/CALM1
peak area (WT)
C
TnSSBP1 - cleavage with Asp-N protease
MTS
MFPALRIPHILRQITGFQHLHSSATQHSTQKQEKTINQVTLLGRVGADPQKRGS
EEHPVINFPLATHFSYKYESGDVLQRTDWHRISIFKPGLRDTVYKFLKKGHRVY
ITGKLSYGEVKMDDGQVRTASTIIADDVIFFQNAPPEH
intensity (counts x10³)
m/z
TnACADS - cleavage with trypsin protease
MTS
MVTSVLLQSSKLSSYSRQCLKAVSQRRTFTSQLTEQQQAVQELARNFAQEHLKPNAA
KYDKEGRFPFDSIKGLTDLGLMGACVDESYNGMGLDYLSLALAVEEVSRGCASTGMI
LSIHNFLYANLVNEKGSPEQKEVFLKNFTKGSLGCFALSEPGAGTDVAGIRTTARLE
GDHWVLNGKKSWVTTAIEGSAIVVFATVDPELKHKGIACFLVPIETEGVFRGK...
intensity (counts x10³)
m/z
TnNDUFA5 - cleavage with Lys-C protease
likely MTS
MGMLKRTTGLVGMAVAPNAHHTLGALYGKILRVLQKMPEKAAYRKYTEQIVRE
RAAVLKQTKDVYEIETKINCGQAEELIIQAENELNLARKMLNWKPWEPLIAKP
PKGQWEWPPTKA
intensity (counts x10³)
m/z
TnMRPS17 - cleavage with Lys-C protease
likely MTS
MAcARNVVETARKFLLLGQCVPTVKQNAAKIRVKRLELDENLLMYFRKDEFYYC
HDPQKVCKTGDIVLIQSLPQKLTKLITHEVKEVVYPFGDITDPITGKKVAKER
YREDMERQAEIYGKLDSTFDYEKAPSRGWQDGKKDFTSKPTYTKFHVFDENDP
YAI
intensity (counts x10³)
m/z

Fig. S7 – Substrate recognition by the UBR4 complex. (A) Comparison of the human UBR4 complex with other giant E3 ligases. The 26S proteasome is used as a size reference. Catalytic domains and putative substrate receptor domains are indicated in red and cyan, respectively. Models used are: HUWE1 (pdb_00007JQ9), UBR5 (pdb_00008EWI), BIRC6 (pdb_00008ATU) and the proteasome (pdb_00006J2X). **(B)** MS analysis of co-purified insect cell proteins in the WT HsUBR4 complex and the ΔBS2 and ΔUBR domain variants. Peak intensity in the WT complex was plotted against the $\log_2$(fold change) of the WT versus the indicated domain deleted complex to identify which co-purified proteins are lost upon domain deletion. Complex components are shown in blue and mitochondrial proteins are shown in red. (**C**) Four insect cell proteins enriched in the WT compared to ΔZZ-DZB complex are shown with their full amino acid sequences. Native N-termini were found using semi-proteolytic specificity searches to identify non-proteolytic N-termini. The protease used to generate peptides is indicated. Fragment spectra are shown for the identified N-terminal peptides. The MTS and predicted cleavage site for TnSSBP1 and TnACADS was identified using the TargetP 2.0 server. TnNDUFA5 and TnMRPS17 N-terminal MTS sequences were predicted by MitoProt II and the iMTS servers but are not predicted to be cleaved. Therefore, all four proteins probably leaked out of mitochondria after import possibly due to damage to mitochondria either during baculovirus infection/expression or cell lysis.

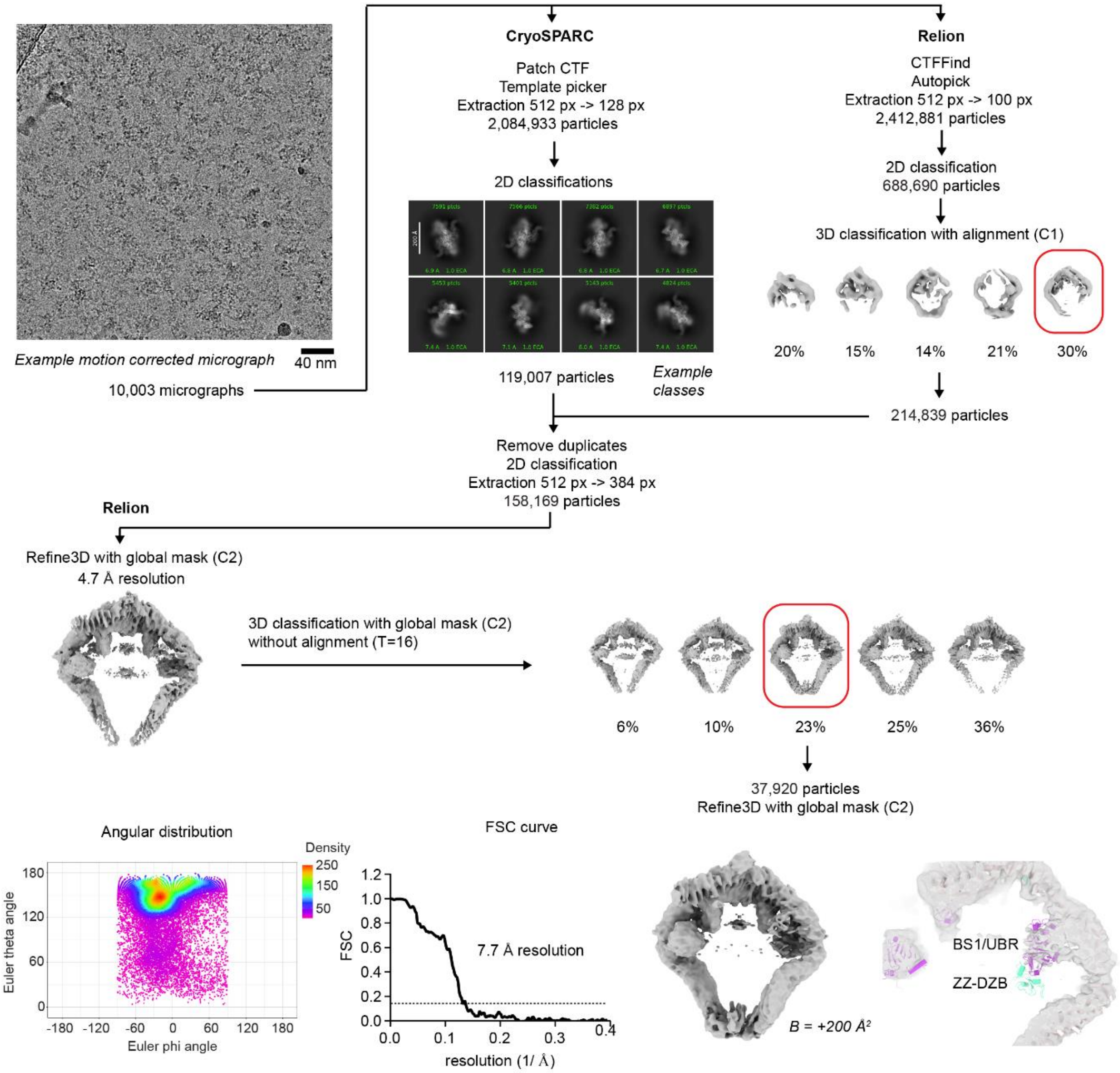

Fig. S8 – Cryo-EM processing pipeline for the ΔZZ-DZB HsUBR4 complex. The same masks were used as for the refinement of the WT complex with a global mask.

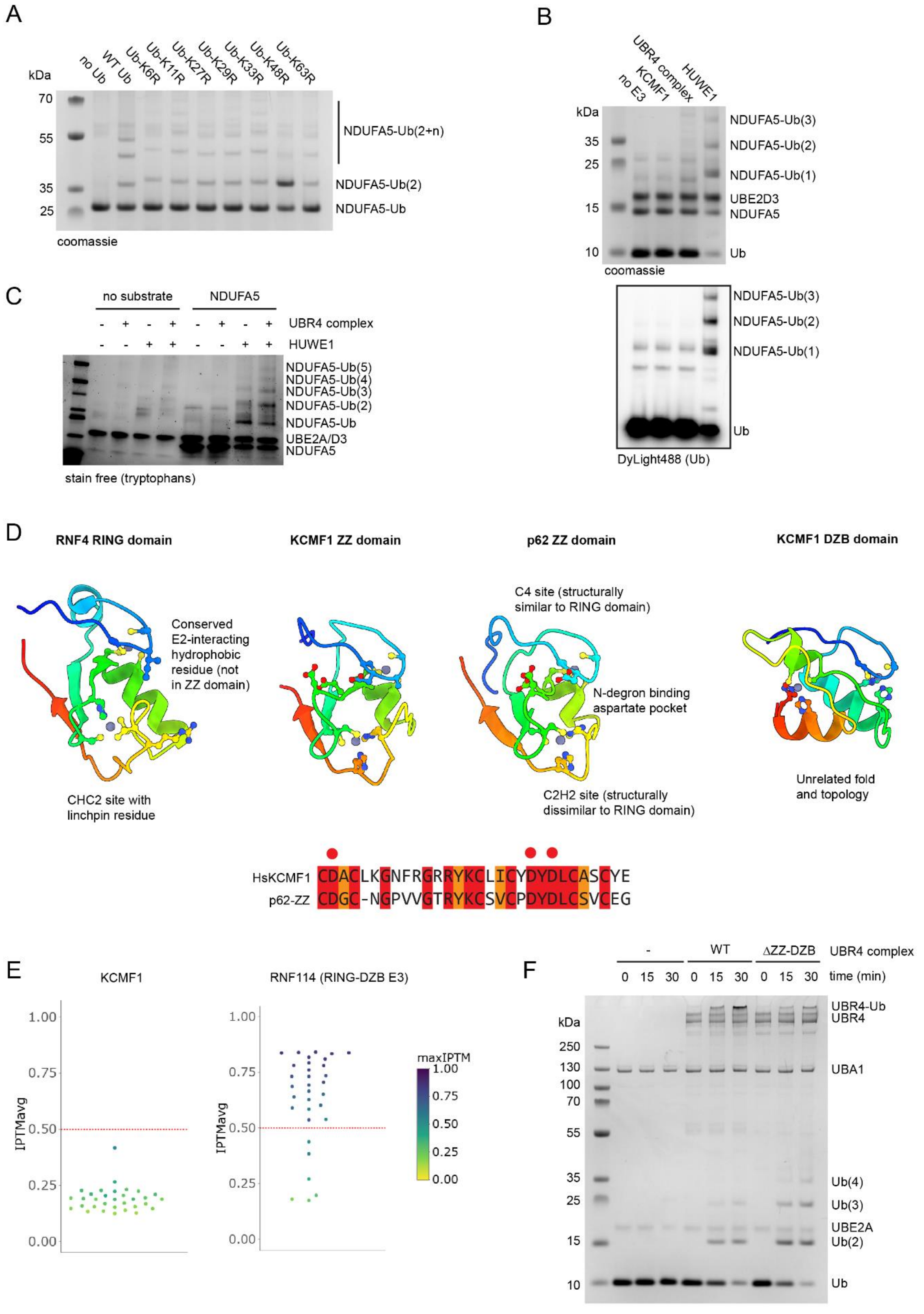
A
kDa
no Ub
WT Ub
Ub-K6R
Ub-K11R
Ub-K27R
Ub-K29R
Ub-K33R
Ub-K48R
Ub-K63R
70
55
35
25
NDUFA5-Ub(2+n)
NDUFA5-Ub(2)
NDUFA5-Ub
coomassie
B
no E3
KCMF1
UBR4 complex
HUWE1
kDa
35
25
15
10
NDUFA5-Ub(3)
NDUFA5-Ub(2)
NDUFA5-Ub(1)
UBE2D3
NDUFA5
Ub
coomassie
NDUFA5-Ub(3)
NDUFA5-Ub(2)
NDUFA5-Ub(1)
Ub
DyLight488 (Ub)
C
no substrate
NDUFA5
UBR4 complex
HUWE1
NDUFA5-Ub(5)
NDUFA5-Ub(4)
NDUFA5-Ub(3)
NDUFA5-Ub(2)
NDUFA5-Ub
UBE2A/D3
NDUFA5
stain free (tryptophans)
D
RNF4 RING domain
KCMF1 ZZ domain
p62 ZZ domain
KCMF1 DZB domain
Conserved E2-interacting hydrophobic residue (not in ZZ domain)
CHC2 site with linchpin residue
C4 site (structurally similar to RING domain)
N-degron binding aspartate pocket
C2H2 site (structurally dissimilar to RING domain)
Unrelated fold and topology
HsKCMF1 CDACLKGNFRGRRYKCLICYDYDLCASCYE
p62-ZZ CDGC-NGPVVGTRYKCSVCPDYDLCSVCEG
E
KCMF1
RNF114 (RING-DZB E3)
IPTMavg
maxIPTM
1.00
0.75
0.50
0.25
0.00
F
-
WT
ΔZZ-DZB
UBR4 complex
0 15 30 0 15 30 0 15 30
time (min)
kDa
250
130
100
70
55
35
25
15
10
UBR4-Ub
UBR4
UBA1
Ub(4)
Ub(3)
UBE2A
Ub(2)
Ub

Fig. S9 – E3 ligase properties of the human UBR4 complex. (A) Ubiquitination assay using 150 nM HsUBR4 complex, 0.5 µM UBE2A, 4 µM NDUFA5-Ub and 20 µM of the indicated ubiquitin variants with single lysine to arginine mutations. While the efficiency of ubiquitin usage varies, only for K48R ubiquitin is there a build-up of the NDUFA5-Ub-Ub species due to blocked further chain elongation. **(B)** Ubiquitination assay of 4 µM NDUFA5 with the addition of either HsKCMF1 alone, the HsUBR4 complex or HsHUWE1 at 0.5 µM and 10 µM ubiquitin (1:10 DyLight488-Ub) and 2.5 µM UBE2D3. **(C)** Ubiquitination assay of 4 µM NDUFA5 by either the HsUBR4 complex at 0.1 µM or HsHUWE1 at 0.5 µM or both at these concentrations for 1 hour at 37°C in the presence of 0.5 µM UBE2A, 2.5 µM UBE2D3 and 40 µM ubiquitin. The gel was imaged by Stain-Free fluorescence (tryptophans) to remove the overlapping signal of free ubiquitin chains, which have no tryptophans. **(D)** Structural and sequence analysis of the N-terminal Zn-binding domains of KCMF1. The RING domain of RNF4 (pdb_00004AP4) is included as a reference. The ZZ domain has the same cross-brace topology as a RING domain but is otherwise structurally unrelated while the ZZ domains of KCMF1 and p62 are highly similar. The DZB domain has an unrelated topology and fold. In the sequence alignment below, ZZ domain N-degron binding residues are highlighted **(E)** An AlphaFold2 pull-down screen where an AlphaFold2 prediction was performed for full-length KCMF1 in the presence of every human E2 enzyme, except the E2/E3 hybrids UBE2O and BIRC6. The iPTM score, indicating the confidence of an interaction is plotted for each prediction. As a control, the screen was repeated with the related protein RNF114, which has a DZB domain with an N-terminal RING domain instead of a ZZ domain. **(F)** Ubiquitination activity of the WT and ΔZZ-DZB HsUBR4 complexes in the absence of substrates using 200 nM complex, 0.5 µM UBE2A and 10 µM ubiquitin for the indicated times at 37°C. The ΔZZ-DZB HsUBR4 complex displays stronger free chain formation but weaker auto-ubiquitination.

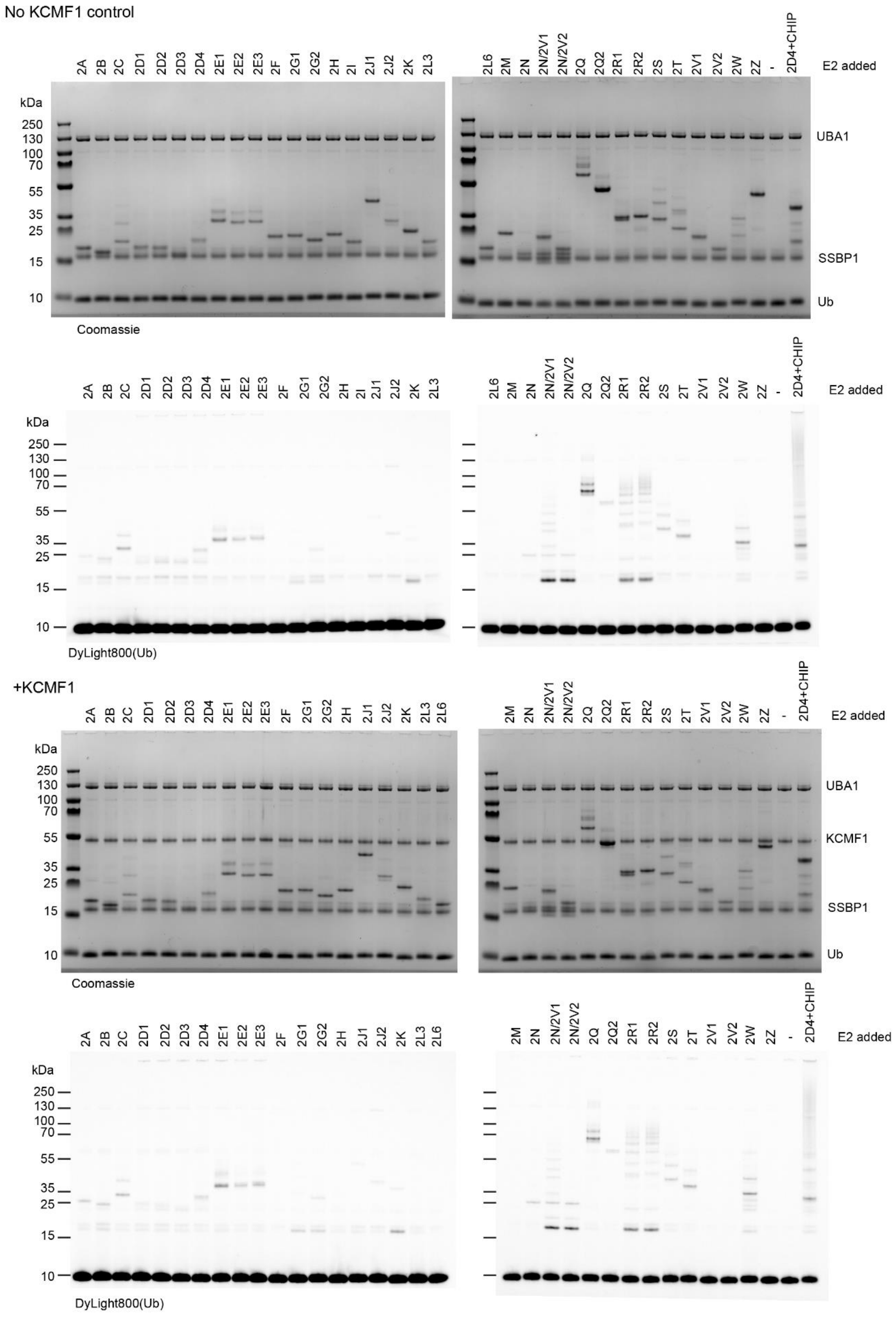
No KCMF1 control
2A 2B 2C 2D1 2D2 2D3 2D4 2E1 2E2 2E3 2F 2G1 2G2 2H 2I 2J1 2J2 2K 2L3
2L6 2M 2N 2N/2V1 2N/2V2 2Q 2Q2 2R1 2R2 2S 2T 2V1 2V2 2W 2Z - 2D4+CHIP
E2 added
kDa
250 130 100 70 55 35 25 15 10
UBA1
SSBP1
Ub
Coomassie
2A 2B 2C 2D1 2D2 2D3 2D4 2E1 2E2 2E3 2F 2G1 2G2 2H 2I 2J1 2J2 2K 2L3
2L6 2M 2N 2N/2V1 2N/2V2 2Q 2Q2 2R1 2R2 2S 2T 2V1 2V2 2W 2Z - 2D4+CHIP
E2 added
kDa
250 130 100 70 55 35 25 15 10
DyLight800(Ub)
+KCMF1
2A 2B 2C 2D1 2D2 2D3 2D4 2E1 2E2 2E3 2F 2G1 2G2 2H 2J1 2J2 2K 2L3 2L6
2M 2N 2N/2V1 2N/2V2 2Q 2Q2 2R1 2R2 2S 2T 2V1 2V2 2W 2Z - 2D4+CHIP
E2 added
kDa
250 130 100 70 55 35 25 15 10
UBA1
KCMF1
SSBP1
Ub
Coomassie
2A 2B 2C 2D1 2D2 2D3 2D4 2E1 2E2 2E3 2F 2G1 2G2 2H 2J1 2J2 2K 2L3 2L6
2M 2N 2N/2V1 2N/2V2 2Q 2Q2 2R1 2R2 2S 2T 2V1 2V2 2W 2Z - 2D4+CHIP
E2 added
kDa
250 130 100 70 55 35 25 15 10
DyLight800(Ub)

Fig. S10 – Activity of KCMF1 in an E2 screen. Ubiquitination assays with 10 μM ubiquitin (1:10 DyLight800-Ub), 4 μM TnSSBP1, 0.25 μM UBA1, 2.5 μM of the indicated E2 enzymes, with or without 3 μM HsKCMF1 were performed for 1 hour at 30°C. The E3 ligase CHIP in the presence of UBE2D4 was used as a positive control resulting in a ubiquitin smear up to the well in the DyLight800 channel. Addition of KCMF1 results in no obvious differences.

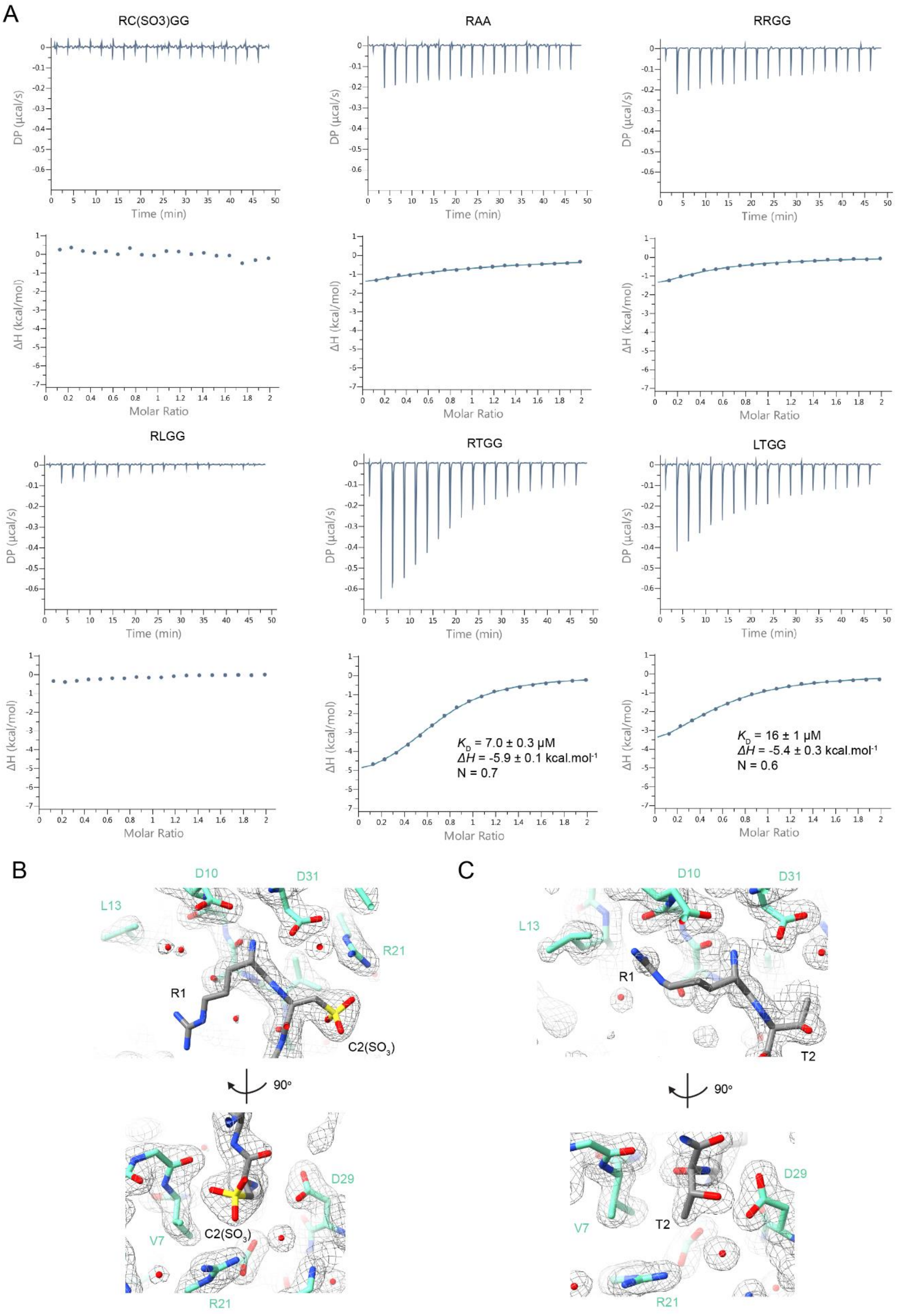
A
RC(SO3)GG
RAA
RRGG
RLGG
RTGG
LTGG
DP (µcal/s)
Time (min)
ΔH (kcal/mol)
Molar Ratio
K_D = 7.0 ± 0.3 µM
ΔH = -5.9 ± 0.1 kcal.mol^{-1}
N = 0.7
K_D = 16 ± 1 µM
ΔH = -5.4 ± 0.3 kcal.mol^{-1}
N = 0.6
B
C
D10
D31
L13
R21
R1
C2(SO$_3$)
T2
90°
D29
V7

Fig. S11 – N-degron binding activity of the ZZ-DZB domains. (A) Isothermal titration calorimetry measurements were performed using 50 μM GST-ZZ-DZB in the cell and 500 μM of the indicated peptides in the syringe. Two titrations could be confidently fitted with a 1:1 binding equation and fitted parameters are shown on these curves. **(B)** Detailed views of the $^{N}RC(SO_3)K$ peptide complex with the KCMF1 ZZ domain showing the 2Fo-Fc map at 1σ. **(C)** Detailed views of the $^{N}RTGG$ peptide complex with the KCMF1 ZZ domain showing the 2Fo-Fc map at 1σ.

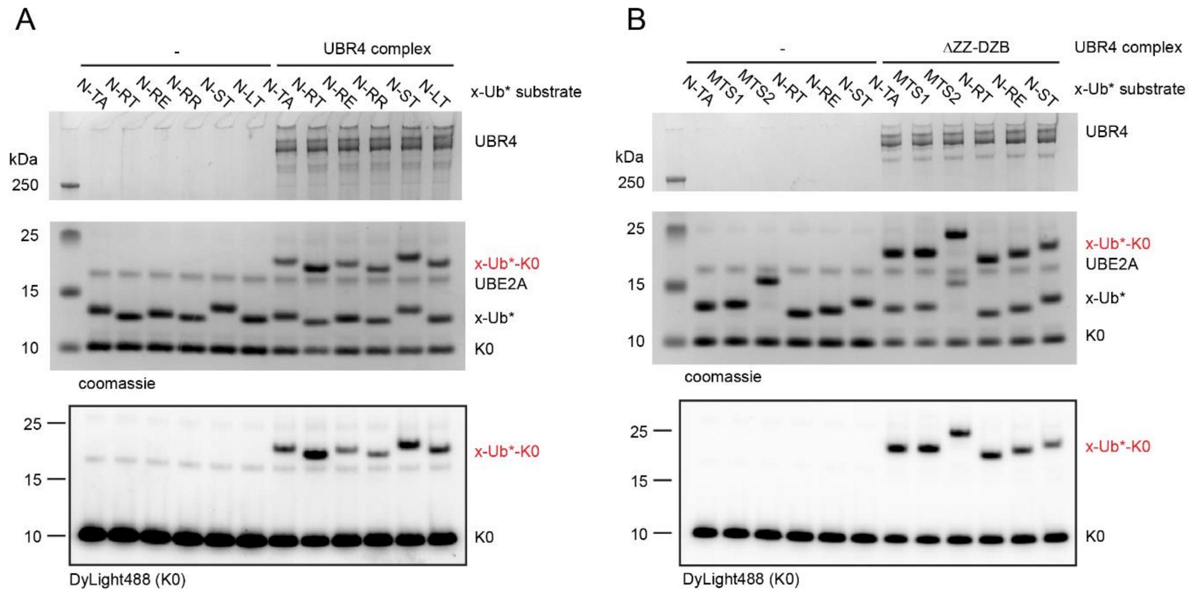

Fig. S12 – Activity of the UBR4 complex against N-degron fused Ub*. The gels corresponding to the quantified data shown in **Fig. 3F** (**A**) and **Fig. 3G** (**B**).

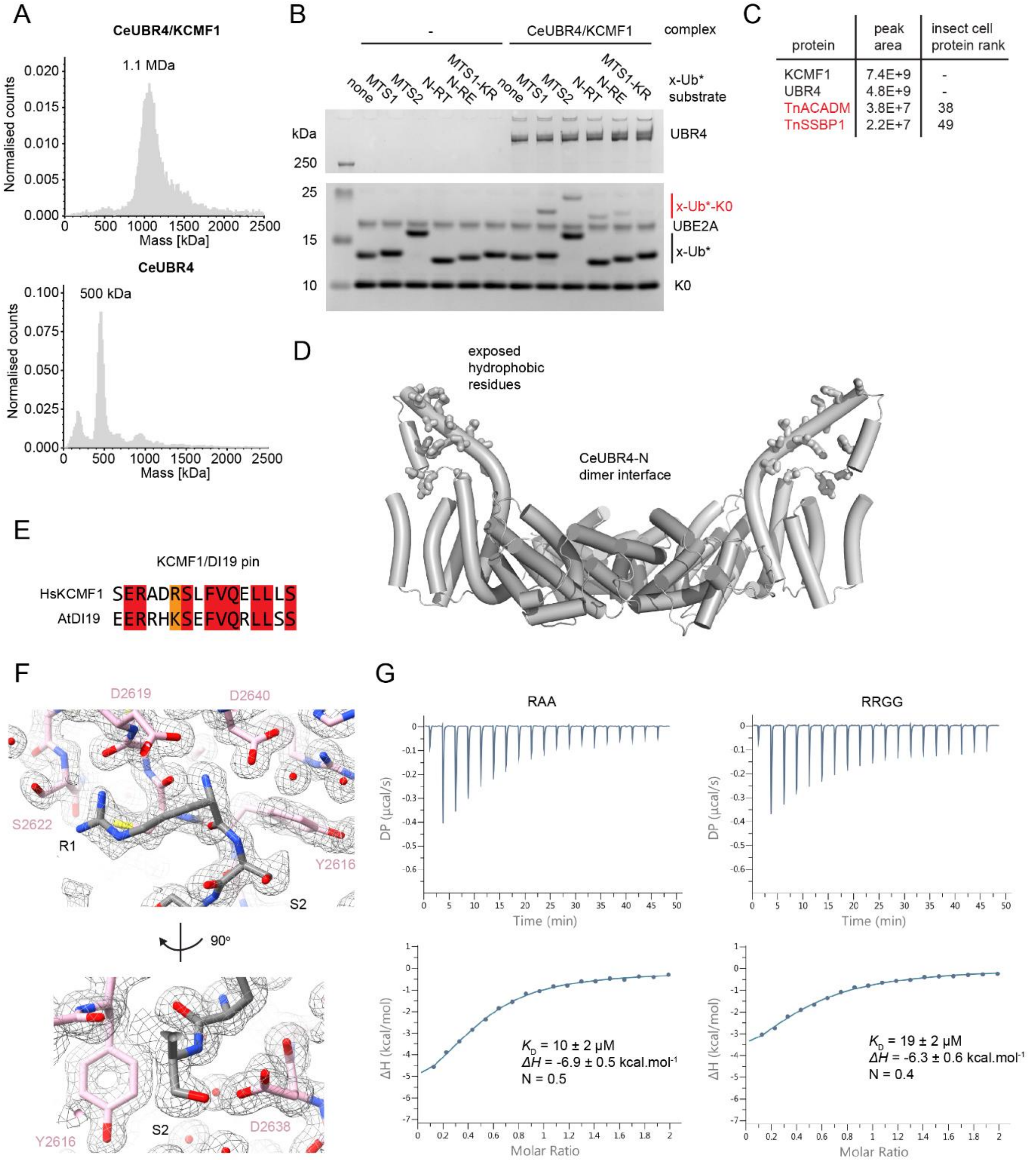

Fig. S13 – Structural and biochemical analysis of orthologues of the UBR4 complex. **(A)** Mass photometry histograms showing the molecular weight distribution of the CeUBR4 complex. The CeUBR4 expressed alone is included as a reference for the molecular weight of monomeric CeUBR4. **(B)** E4 ligase assay using 200 nM CeUBR4 complex with 500 nM HsUBE2A and 250 nM HsUBA1 and the indicated Ub* substrates for 45 minutes at 37°C. **(C)** Summary of MS data of purified CeUBR4 complex showing rank of co-purified insect cell proteins by peak area with mitochondrial proteins coloured in red. **(D)** AlphaFold3 model of the CeUBR4 N-terminal dimer interface. All the exposed hydrophobic residues on the two helices which enter into the central arena are shown in stick representation. These helices were not fully

resolved in the cryo-EM structure due to flexibility. **(E)** Sequence alignment of the pin region from HsKCMF1 and AtDI19. **(F)** Detailed view of the ^{N}RS N-terminus (grey) interaction with the AtUBR4 ZZ domain (pink) showing the 2Fo-Fc map at 1σ. **(G)** Isothermal titration calorimetry measurements were performed using 50 μM GST-ZZ-DZB in the cell and 500 μM of the indicated peptides in the syringe. Titration curves were fitted with a 1:1 binding equation and fitted parameters are shown on these curves.

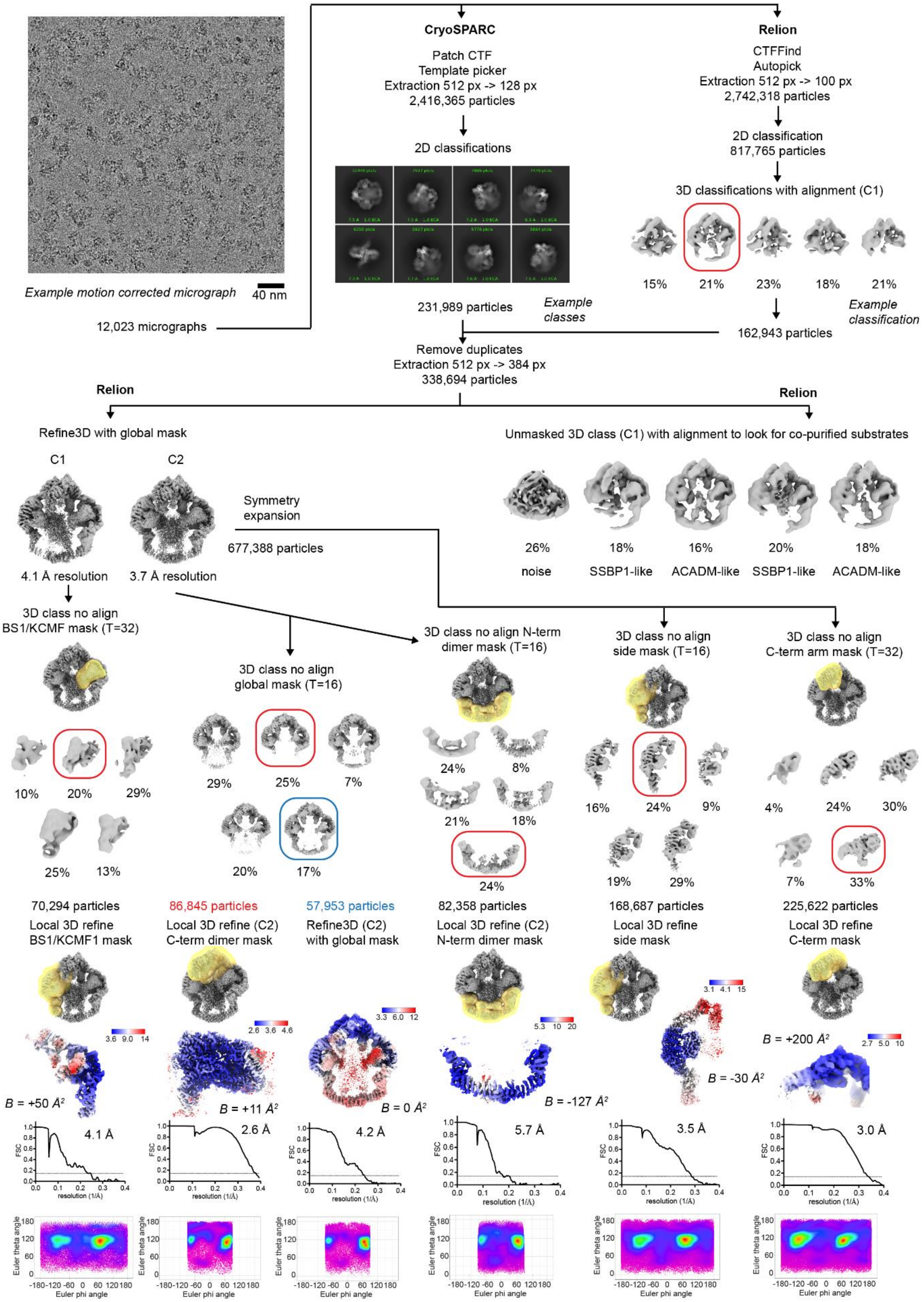

CryoSPARC
Patch CTF
Template picker
Extraction 512 px -> 128 px
2,416,365 particles
2D classifications
Relion
CTFFind
Autopick
Extraction 512 px -> 100 px
2,742,318 particles
2D classification
817,765 particles
3D classifications with alignment (C1)
15%
21%
23%
18%
21%
Example classification
Example motion corrected micrograph
40 nm
12,023 micrographs
231,989 particles
Example classes
162,943 particles
Remove duplicates
Extraction 512 px -> 384 px
338,694 particles
Relion
Relion
Refine3D with global mask
C1
C2
Symmetry expansion
677,388 particles
4.1 Å resolution
3.7 Å resolution
Unmasked 3D class (C1) with alignment to look for co-purified substrates
26%
18%
16%
20%
18%
noise
SSBP1-like
ACADM-like
SSBP1-like
ACADM-like
3D class no align BS1/KCMF mask (T=32)
3D class no align global mask (T=16)
3D class no align N-term dimer mask (T=16)
3D class no align side mask (T=16)
3D class no align C-term arm mask (T=32)
10%
20%
29%
25%
13%
29%
25%
7%
20%
17%
24%
8%
21%
18%
24%
16%
24%
9%
19%
29%
4%
24%
30%
7%
33%
70,294 particles
Local 3D refine
BS1/KCMF1 mask
86,845 particles
Local 3D refine (C2)
C-term dimer mask
57,953 particles
Refine3D (C2)
with global mask
82,358 particles
Local 3D refine (C2)
N-term dimer mask
168,687 particles
Local 3D refine
side mask
225,622 particles
Local 3D refine
C-term mask
B = +50 Å²
B = +11 Å²
B = 0 Å²
B = -127 Å²
B = -30 Å²
B = +200 Å²
4.1 Å
2.6 Å
4.2 Å
5.7 Å
3.5 Å
3.0 Å
FSC
resolution (1/Å)
Euler theta angle
Euler phi angle

Fig. S14 – Cryo-EM processing pipeline for the CeUBR4 complex. Analysis of the different substrate bound states is shown in the center-right of the pipeline.

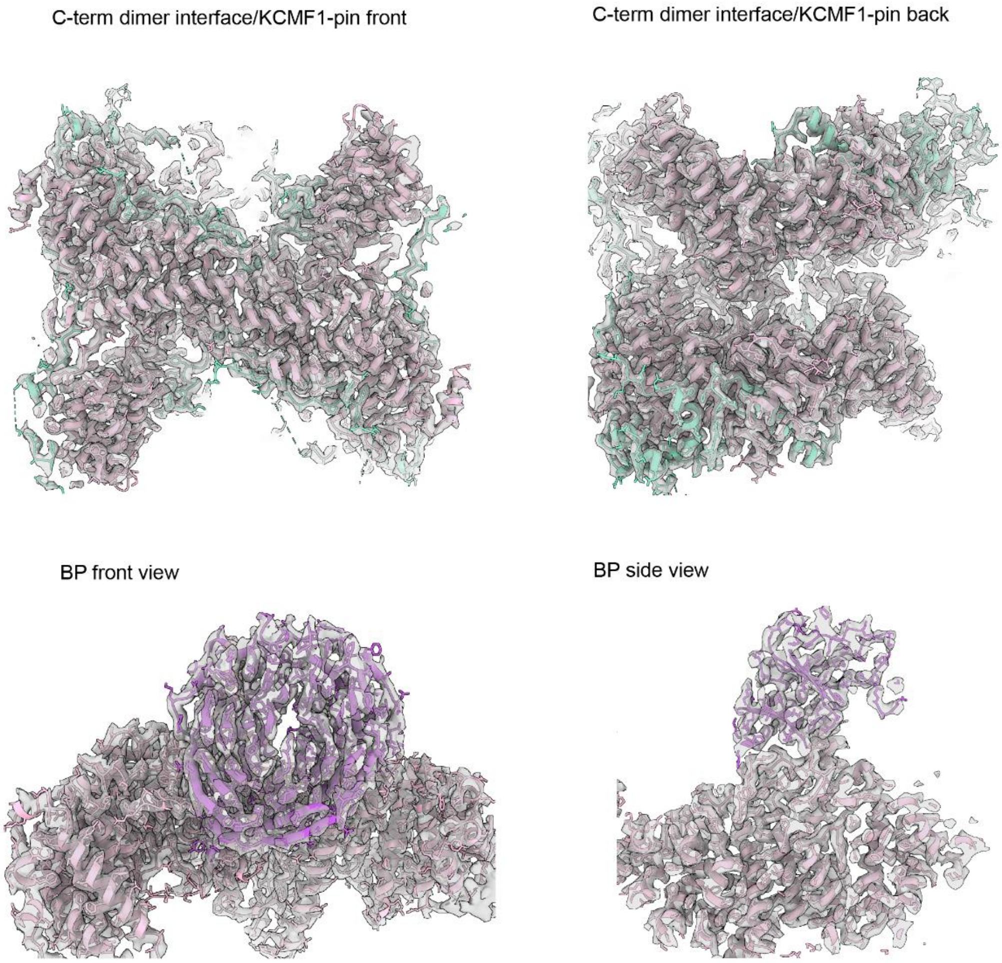

Fig. S15 – Example map to model fits for different regions of the CeUBR4 complex structure which were of sufficient resolution to build de novo using ModelAngelo.

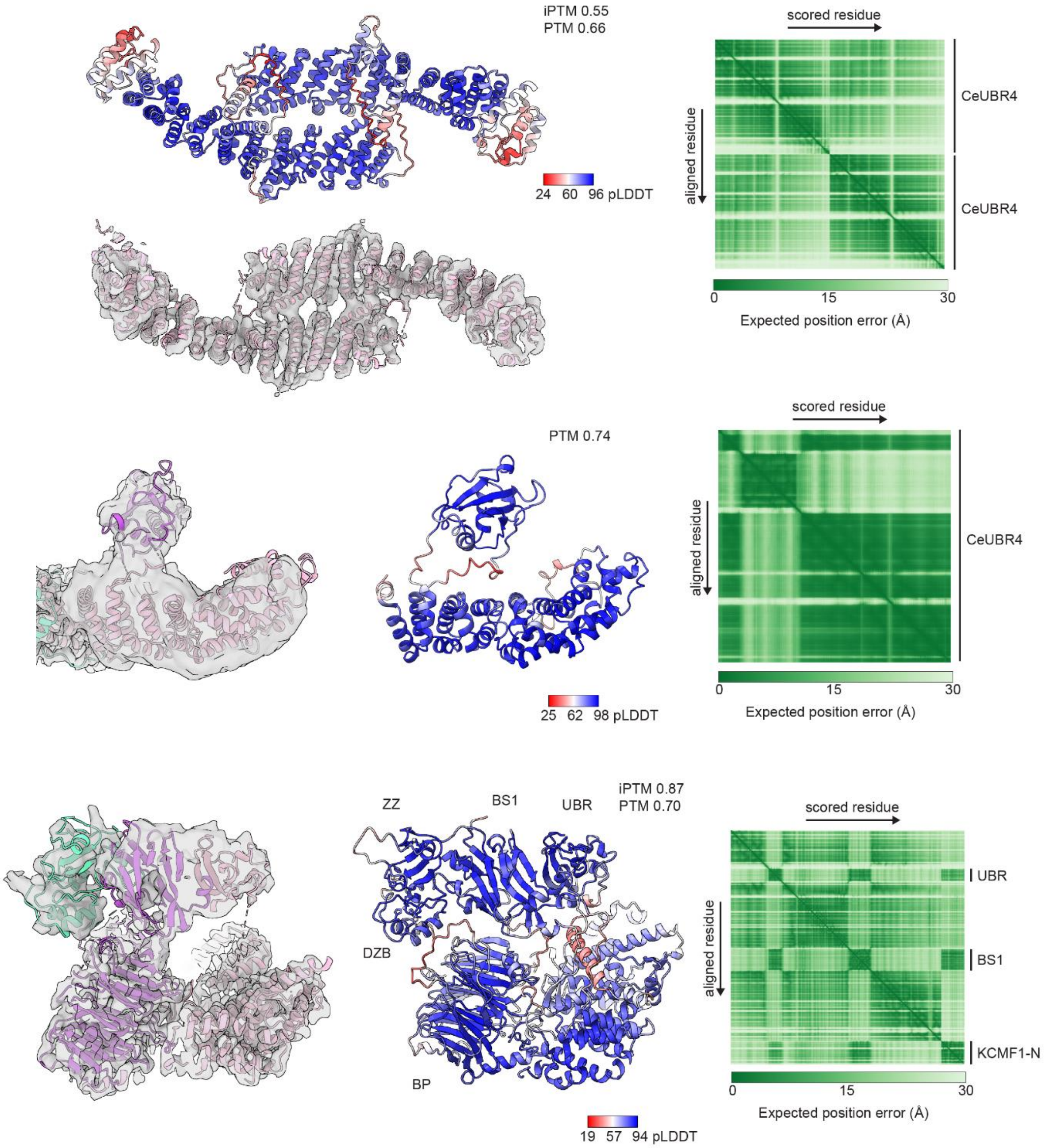

Fig. S16 – AlphaFold3 models for sections of the CeUBR4 complex with map resolutions too low for de novo modelling. The pLDDT, PAE, PTM and iPTM scores are shown as indicators of confidence for each AlphaFold3 prediction as well as the model to map fit of these models into the experimental cryo-EM density.

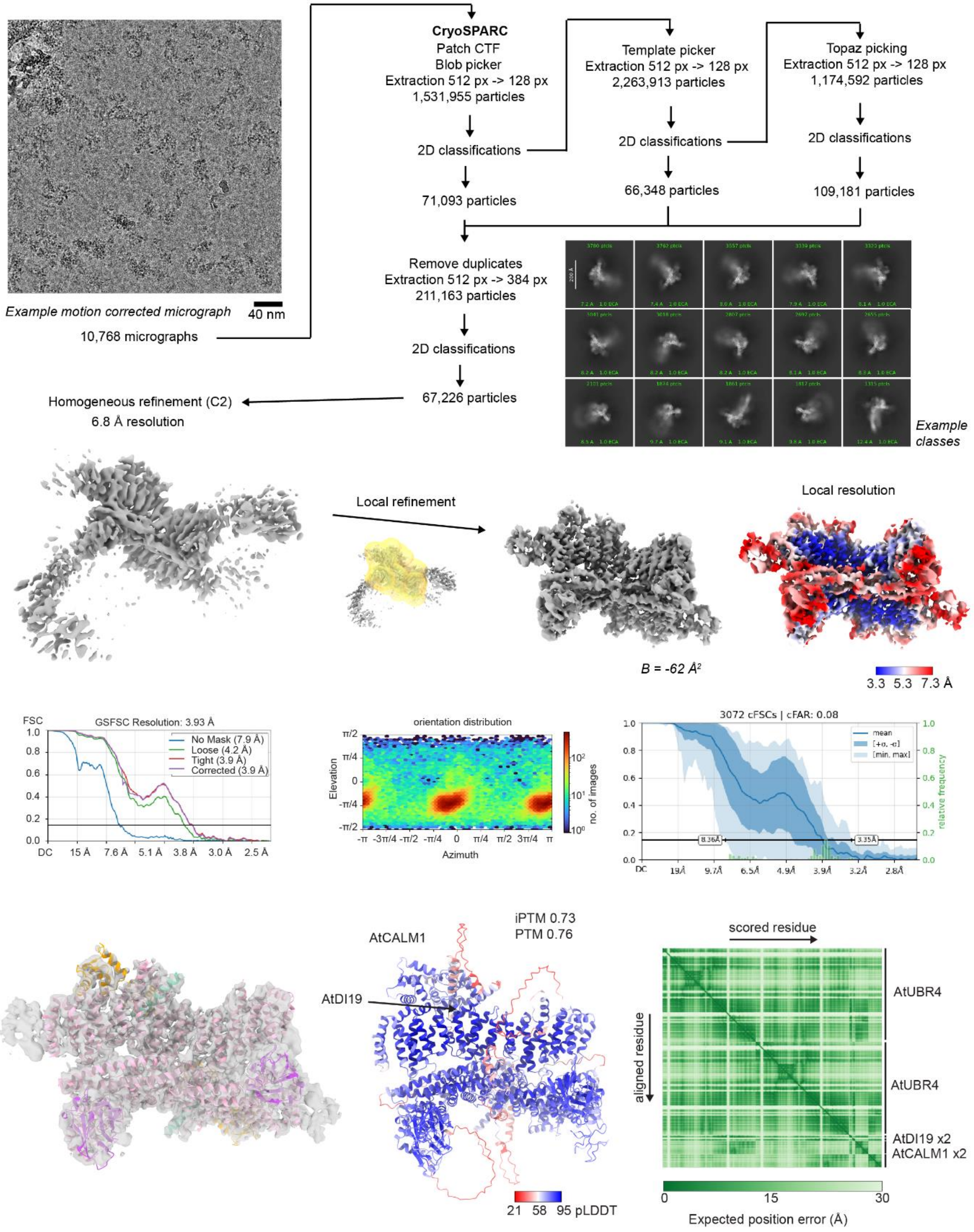

Fig. S17 – Cryo-EM processing pipeline for the C-terminal dimerization core of the AtUBR4 complex. In 2D classes the closed ring shape formed by the UBR4 N-termini is faintly visible as well as the C-terminal Armadillo extensions but these were too flexible for even moderate resolution 3D reconstruction. At the bottom of the figure, details of the AlphaFold3 prediction used to model the complex are shown.

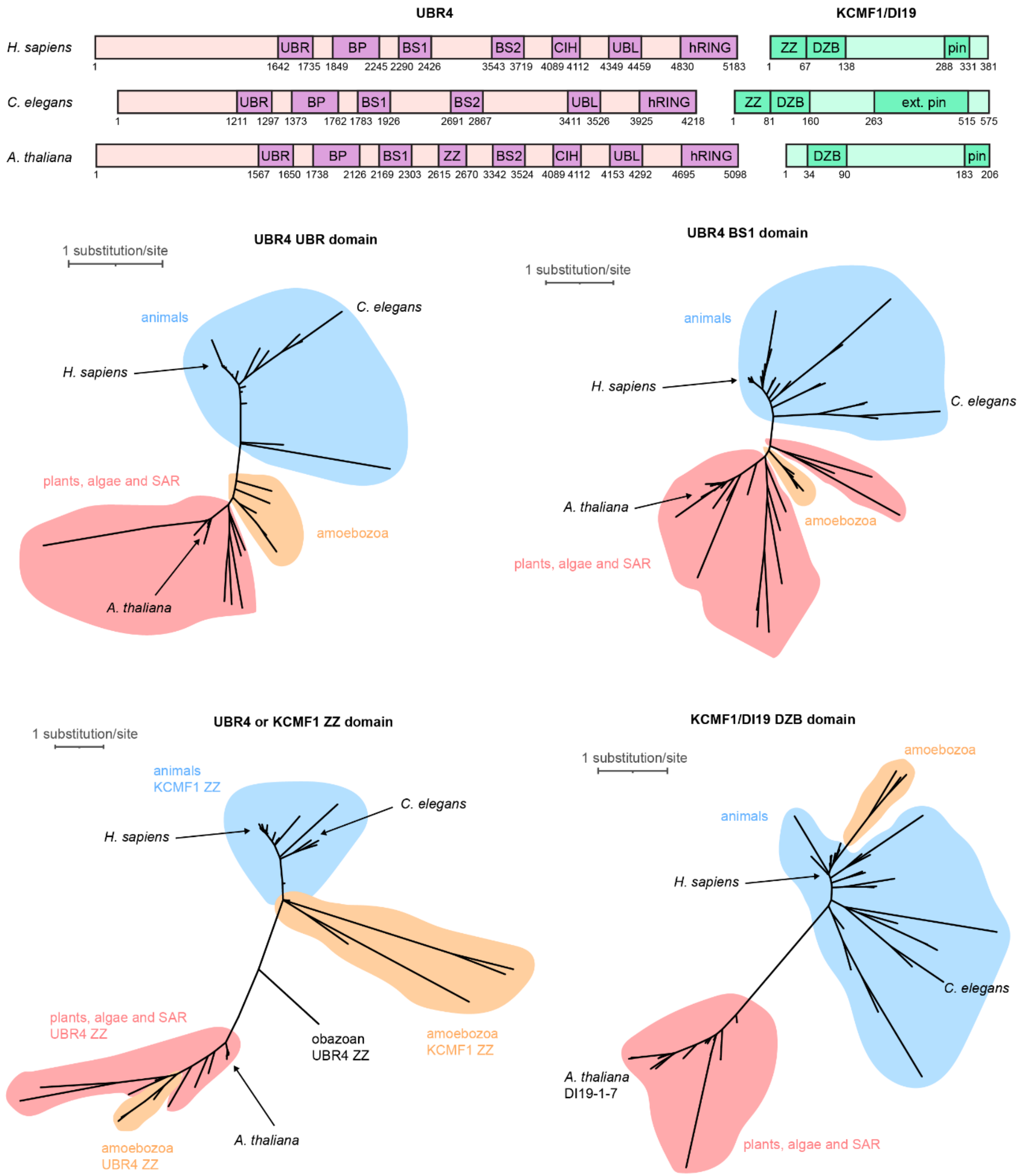

Fig. S18 – Phylogenetic analysis of UBR4 and KCMF1/DI19. In the upper panel, the domain architectures of the three UBR4/KCMF1 complexes we structurally characterized are shown. In the lower panels are phylogenetic trees of domains calculated from a selection of UBR4 and KCMF1 homologues representing diverse phyla. For simplification of the ZZ and DZB domain trees, we only used sequences corresponding from bona fide UBR4 and KCMF1 orthologues. It is likely that ZZ and DZB domains from different proteins are more related to the two distinct groupings observed here (plants/algae/SAR vs metazoan) than these grouping are to each other. Some amoebozoa have a plant-related ZZ domain in their UBR4 orthologue and a

metazoan-related ZZ domain in their KCMF1 orthologue, suggesting that the two domains may have a distinct evolutionary origin.

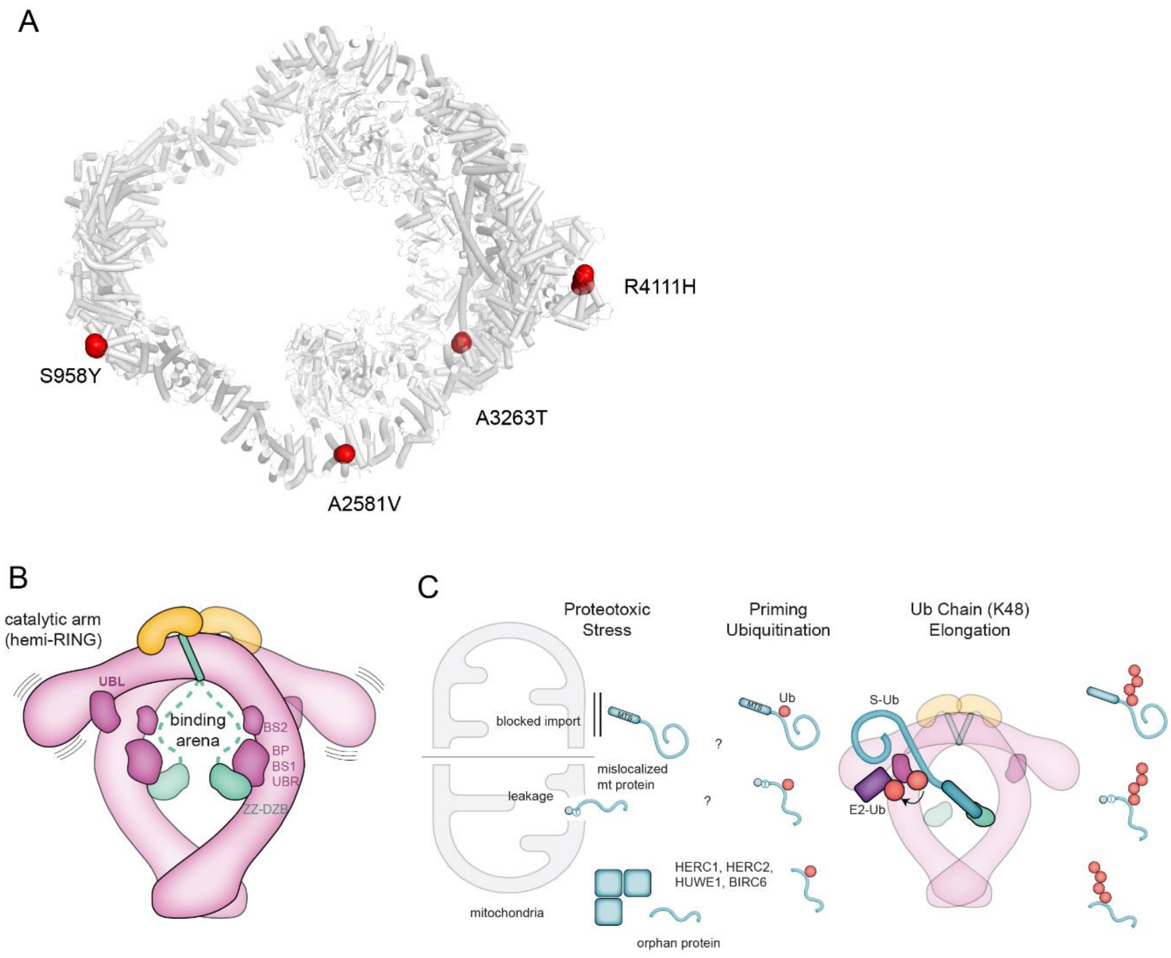

Fig. S19 – Cellular function of the UBR4 complex (A) UBR4 patient mutations within the Armadillo repeats. Mutated residues are shown as red spheres. **(B)** Schematic of the main functional domains of the UBR4 complex **(C)** Model for the role of the E4 activity of the UBR4 complex in cells.

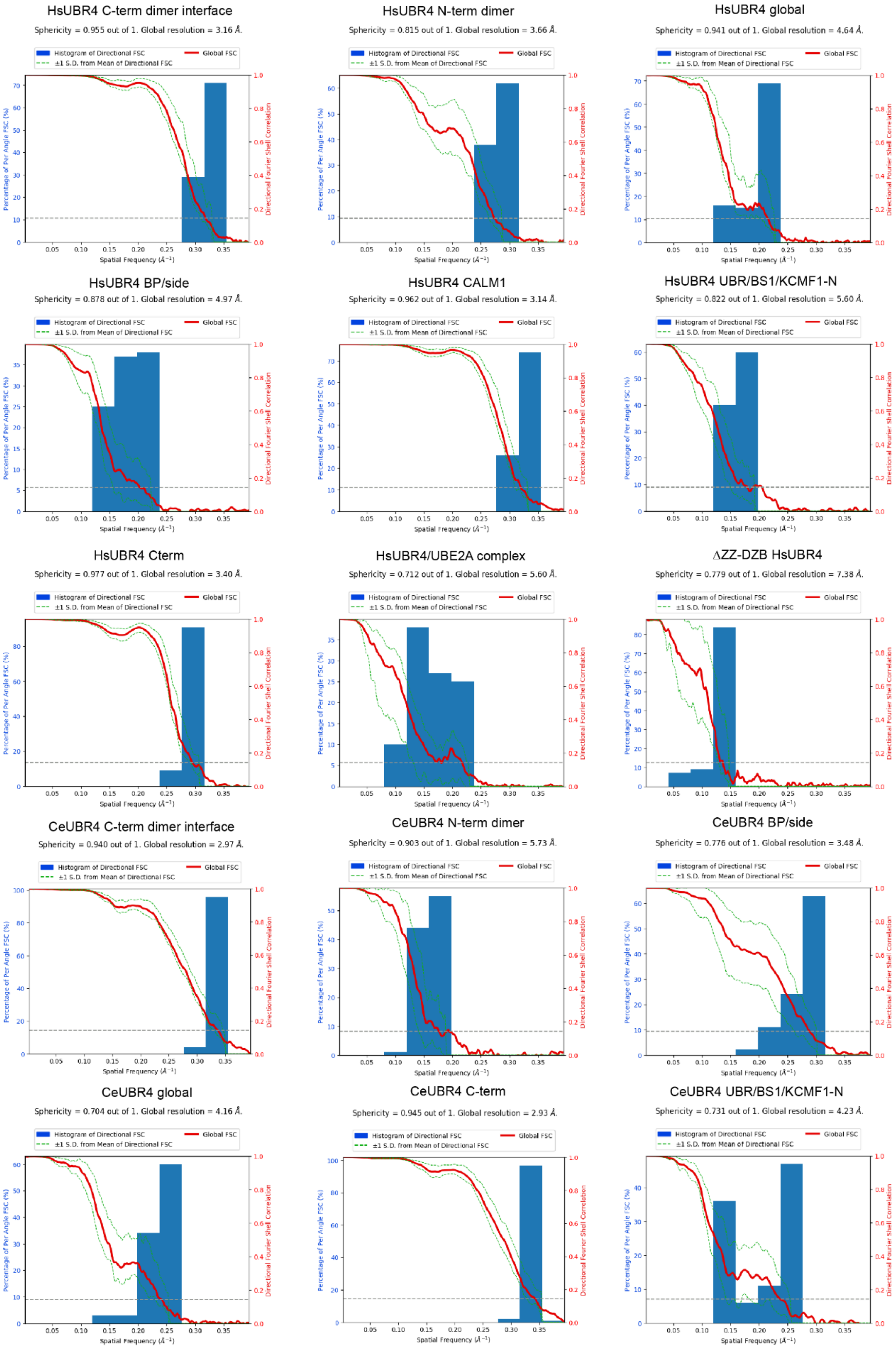
HsUBR4 C-term dimer interface
Sphericity = 0.955 out of 1. Global resolution = 3.16 Å.
HsUBR4 N-term dimer
Sphericity = 0.815 out of 1. Global resolution = 3.66 Å.
HsUBR4 global
Sphericity = 0.941 out of 1. Global resolution = 4.64 Å.
HsUBR4 BP/side
Sphericity = 0.878 out of 1. Global resolution = 4.97 Å.
HsUBR4 CALM1
Sphericity = 0.962 out of 1. Global resolution = 3.14 Å.
HsUBR4 UBR/BS1/KCMF1-N
Sphericity = 0.822 out of 1. Global resolution = 5.60 Å.
HsUBR4 Cterm
Sphericity = 0.977 out of 1. Global resolution = 3.40 Å.
HsUBR4/UBE2A complex
Sphericity = 0.712 out of 1. Global resolution = 5.60 Å.
ΔZZ-DZB HsUBR4
Sphericity = 0.779 out of 1. Global resolution = 7.38 Å.
CeUBR4 C-term dimer interface
Sphericity = 0.940 out of 1. Global resolution = 2.97 Å.
CeUBR4 N-term dimer
Sphericity = 0.903 out of 1. Global resolution = 5.73 Å.
CeUBR4 BP/side
Sphericity = 0.776 out of 1. Global resolution = 3.48 Å.
CeUBR4 global
Sphericity = 0.704 out of 1. Global resolution = 4.16 Å.
CeUBR4 C-term
Sphericity = 0.945 out of 1. Global resolution = 2.93 Å.
CeUBR4 UBR/BS1/KCMF1-N
Sphericity = 0.731 out of 1. Global resolution = 4.23 Å.
Histogram of Directional FSC
Global FSC
±1 S.D. from Mean of Directional FSC
Percentage of Per Angle FSC (%)
Directional Fourier Shell Correlation
Spatial Frequency (Å⁻¹)

Fig. S20 – 3D FSC curves for all cryo-EM maps associated with this manuscript refined in Relion. 3D FSC curves calculated by the 3D FSC Server (3dfsc.salk.edu) are shown.

Uncropped gels

Fig. 2A

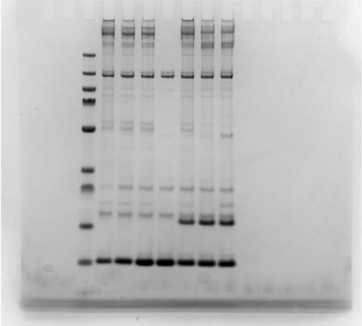

Fig. 2D

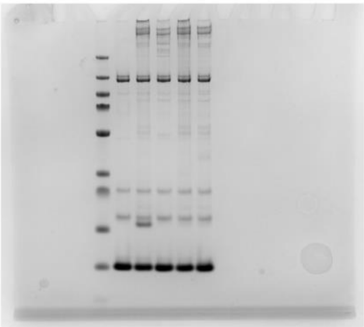

Fig. 3C

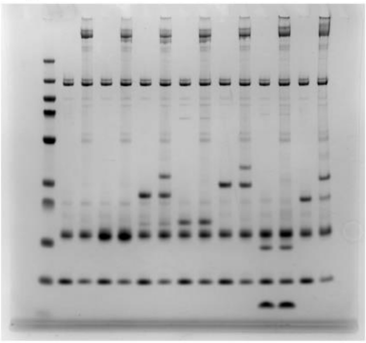

Fig. 3D

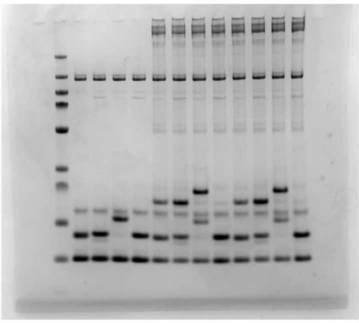

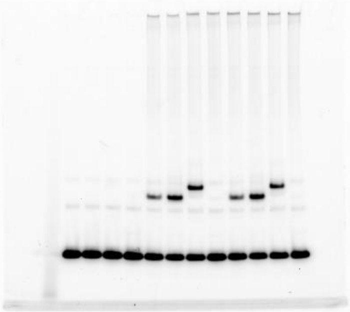

Fig. S5B

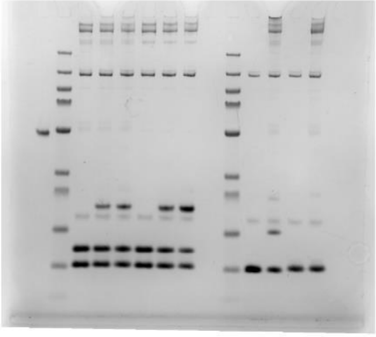

Fig. S9A

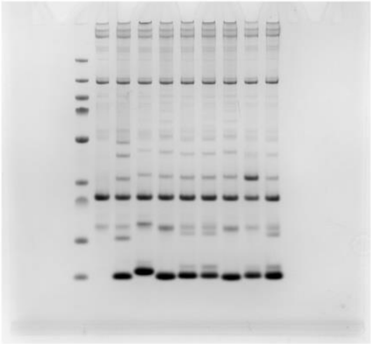

Fig. S9B

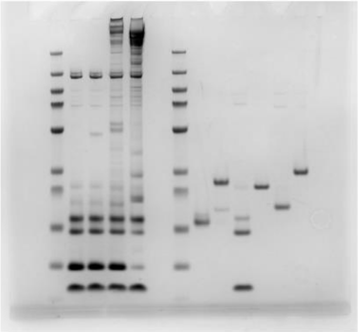

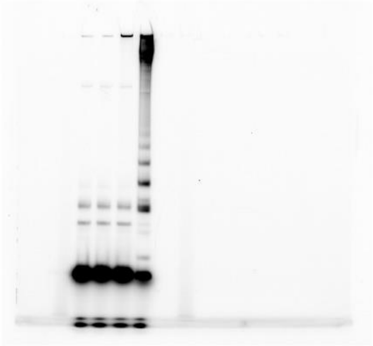

Fig. S9C

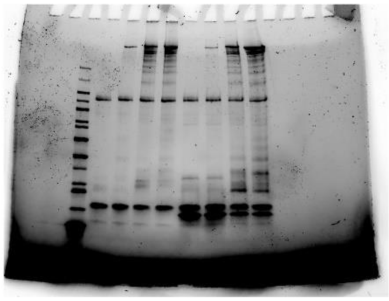

Fig. S12A

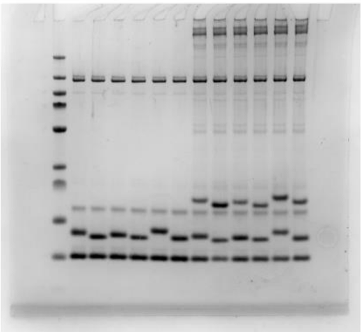

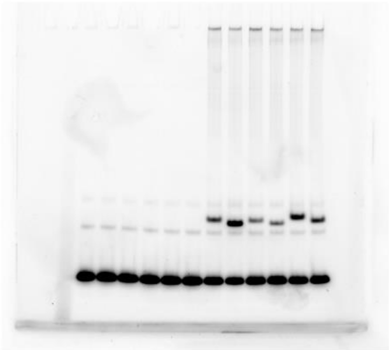

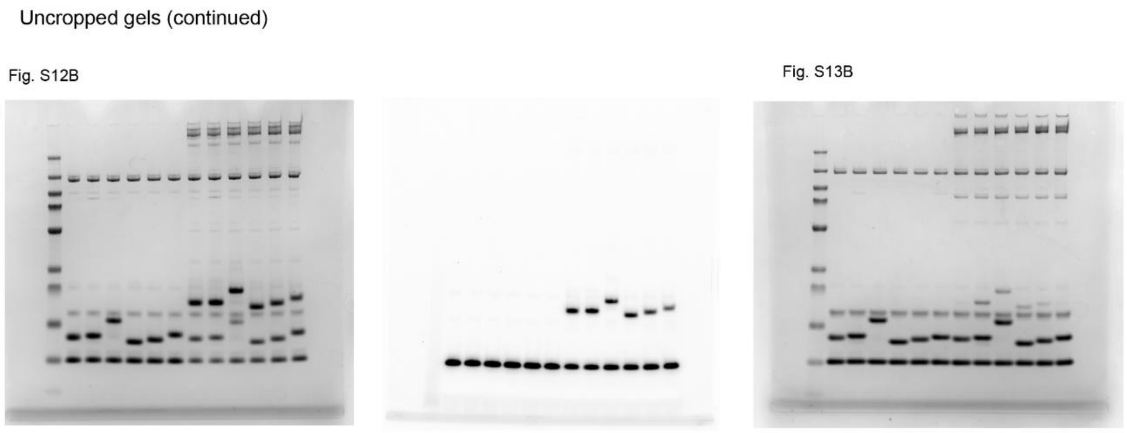

Fig. S21 – Uncropped gels

	HsUBR4 complex (global) EMD-52491	HsUBR4 complex (C-term dimer interface) PDB_ 00009QWS EMD-53426	HsUBR4 complex (N-term dimer interface) PDB_ 00009QWX EMD-53430	HsUBR4 complex (side)* PDB_ 00009QWZ EMD-53431	HsUBR4 complex (BS1/UBR/KCMF1-N) EMD-52494	HsUBR4 complex (CALM1) PDB_ 00009QWU EMD-53428	HsUBR4 complex (C-term)* PDB_ 00009QX0 EMD-53432
Magnification				130,000x			
Microscope				Titan Krios G4			
Detector				Falcon 4			
No. of frames				40			
Energy filter slit width (eV)				10			
Automation software				EPU			
Micrographs (no.)				34,647			
Voltage (kV)				300			
Electron exposure (e^-/Å^2)				50			
Defocus range (µm)				-0.8 – -2.0			
Pixel size (Å)				0.951->1.27			
Symmetry imposed	C2	C2	C2	C1 (symmetry expanded)	C1 (symmetry expanded)	C1 (symmetry expanded)	C1 (symmetry expanded)
Initial particle images (no.)	6,406,199+ 5,456,481	6,406,199+ 5,456,481	6,406,199+ 5,456,481	6,406,199+ 5,456,481	6,406,199+ 5,456,481	6,406,199+ 5,456,481	6,406,199+ 5,456,481
Final particle images (no.)	78,983	135,406	75,153	325,440	220,038	464,266	339,437
Map resolution (Å)	4.7	3.1	3.6	4.8	5.6	3.1	3.4
FSC threshold	0.143	0.143	0.143	0.143	0.143	0.143	0.143
3DFSC sphericity	0.941	0.955	0.815	0.878	0.822	0.962	0.977
Map resolution range	3.7-26	3.0-13	3.5-9.4	4.5-14	4.3-22	2.9-4.9	3.2-6.2

Estimated accuracy of rotations (°)/ offsets (Å)	0.855/ 0.937	0.746/ 0.673	0.688/ 0.931	1.80/ 1.78	2.21/ 1.81	0.994/ 0.796	1.36/ 1.13
Initial model used	No model	ModelAngelo	ModelAngelo	AlphaFold3	No model	AlphaFold3	AlphaFold3
Atomic Refinement package		PHENIX	PHENIX	PHENIX		PHENIX	PHENIX
Model resolution (Å)		3.3	4.0	7.1		3.3	5.9
FSC threshold		0.5	0.5	0.5		0.5	0.5
CCvolume/CCmask		0.81/0.83	0.79/0.80	0.75/0.75		0.82/0.84	0.85/0.85
Map sharpening *B* factor ($Å^2$)	-76	-32	0	-200	0	-30	+200
Non-hydrogen atoms		18,574	20,114	7,115		17,031	11,445
Protein residues		2,414	2,702	1,433		2,198	2,306
Ligands		2xZn^{2+}	-	-		2xZn^{2+}	-
B factors ($Å^2$)							
Protein		73.6	113	270		70.1	400
Ligand		102	-	-		106	-
r.m.s.d.							
Bond lengths (Å)		0.003	0.003	0.004		0.004	0.002
Bond angles (°)		0.553	0.692	0.671		0.583	0.490
MolProbity score		1.37	2.00	1.61		1.39	0.90
Clashscore		4.06	9.24	3.70		4.89	0.60
Poor rotamers (%)		1.74	2.17	0		1.48	0
Ramachandran							
Favored (%)		98.14	96.26	93.01		98.42	96.76
Allowed (%)		1.86	3.59	6.99		1.58	3.24
Disallowed (%)		0	0.15	0		0	0
CaBLAM outliers (%)		0.6	2.00	1.57		0.48	0.73
EMRinger Score		1.66	1.30	*		1.54	*
Average Q-score		0.508	0.396	0.358		0.512	0.399

Table S1 – Cryo-EM data collection and refinement statistics. See Fig. S2 for details. *Models did not include sidechains and so no EMRinger score could be calculated.

	HsUBR4 complex with UBE2A EMD-52488	ZZ-DZB HsUBR4 complex EMD-53425
Magnification	130,000x	130,000x
Microscope	Titan Krios G4	Titan Krios G4
Detector	Falcon 4	Falcon 4
No. of frames	40	40
Energy filter slit width (eV)	10	10
Automation software	EPU	EPU
Micrographs (no.)	20,612	10,003
Voltage (kV)	300	300
Electron exposure (e^-/Å^2)	50	50
Defocus range (μm)	-0.8 – -2.0	-0.8 – -2.0
Pixel size (Å)	0.951->1.27	0.951->1.27
Symmetry imposed	C1 (symmetry expanded)	C2
Initial particle images (no.)	4,484,818+ 3,827,533	2,084,933+ 2,412,881
Final particle images (no.)	154,691	37,920
Map resolution (Å)	5.9	7.7
FSC threshold	0.143	0.143
3DFSC sphericity	0.712	0.779
Map resolution range	4.2-37	5.6-30
Estimated accuracy of rotations (^{o})/ offsets (Å)	1.91/1.87	1.26/1.37

Table S2 – Cryo-EM data collection and refinement statistics (continued). See Figs. S6 and S8 for details.

	R-BIG^{ZZ}	KCMF^{ZZ} + RC$_{O3}$	KCMF^{ZZ} + RT
Data collection			
Space group	C 2 2 2	P 4_3 3 2	P 4_3 3 2
Cell dimensions			
a, *b*, *c* (Å)	79.747, 146.855,	97.592, 97.592,	97.347 97.347 97.347
α, β, γ (°)	90, 90, 90	90, 90, 90	90, 90, 90
Wavelength (Å)	1.00	1.28	1.28
Resolution (Å)	50–1.5 (1.53–1.5)*	30–1.92 (1.99–1.92)*	50–1.71 (1.77–1.71)*
R_{merge}	0.099 (0.728)	0.1232 (2.702)	0.1609 (2.464)
I / σ (I)	25.6 (2.0)	38.10 (2.05)	23.61 (1.52)
Completeness (%)	99.2 (95.8)	99.69 (97.41)	99.53 (98.77)
Redundancy	12.7 (9.2)	75.9 (71.9)	31.2 (16.7)
Beamline	PAL 5C	PAL 5C	PAL 5C
Refinement			
Resolution (Å)	36.71–1.5 (1.55–1.5)*	29.43–1.92 (1.99–1.92)*	34.42–1.71 (1.77–1.71)*
No. reflections	46,148 (4,322)	12,642 (1,203)	17414 (1687)
R_{work}	0.1753 (0.2801)	0.1974 (0.2901)	0.2164 (0.2725)
R_{free}	0.1846 (0.2952)	0.2291 (0.3225)	0.2332 (0.3008)
No. of atoms	1,564	975	957
Macromolecules	1,404	938	914
Hetero-atoms	6 Zn, 3 NI	4 Zn	4 Zn
Waters	151	33	39
Protein residues	177	124	122
B-factors (Å^2)	19.19	48.11	29.66
R.m.s. deviations			
Bond length (Å)	0.009	0.013	0.010
Bond angles (°)	1.21	1.45	1.26
Ramachandran statistics			
Favored (%)	96.49	98.31	98.31

Allowed (%)	3.51	1.69	1.69
Outliers (%)	0.00	0.00	0.00
PDB ID	PDB_00009LGS	PDB_00009JNI	PDB_00009UPZ

Table S3 – Crystallography data collection, phasing, and refinement statistics.

	CeUBR4 complex (global) EMD-52504	CeUBR4 complex (C-term dimer interface) PDB_ 00009QX1 EMD-53433	CeUBR4 complex (N-term dimer interface)* PDB_ 00009QX2 EMD-53434	CeUBR4 complex (side) PDB_ 00009QX5 EMD-53435	CeUBR4 complex (BS1/UBR/KCMF1-N) EMD-52513	CeUBR4 complex (C-term) EMD-52516	AtUBR4 complex (C-term dimer interface)* PDB_ 00009QT9 EMD-53348
Magnification	130,000x						130,000x
Microscope	Titan Krios G4						Titan Krios G4
Detector	Falcon 4						Falcon 4
No. of frames	40						40
Energy filter slit width (eV)	10						10
Automation software	EPU						EPU
Micrographs (no.)	12,023						10,768
Voltage (kV)	300						300
Electron exposure (e^-/Å^2)	50						50
Defocus range (µm)	-0.8 – -2.0						-0.8 – -2.0
Pixel size (Å)	0.951->1.27						0.951->1.27
Symmetry imposed	C2	C2	C2	C1 (symmetry expanded)	C1	C1 (symmetry expanded)	C2
Initial particle images (no.)	2,416,365+ 2,742,318	2,416,365+ 2,742,318	2,416,365+ 2,742,318	2,416,365+ 2,742,318	2,416,365+ 2,742,318	2,416,365+ 2,742,318	1,531,955+ 2,263,913+ 1,174,592
Final particle images (no.)	57,953	86,845	82,358	168,687	70,294	225,622	67,226
Map resolution (Å)	4.2	2.6	5.7	3.5	4.1	3.0	3.9
FSC threshold	0.143	0.143	0.143	0.143	0.143	0.143	0.143
3DFSC sphericity	0.704	0.940	0.903	0.776	0.731	0.945	-
Map resolution range	3.3-25	2.6-13.2	5.2-22	3.1-23	3.6-17	2.7-9.8	3.3-59

Estimated accuracy of rotations (°)/ offsets (Å)	0.883/0.851	0.547/0.507	1.61/1.64	1.03/1.01	1.40/1.29	0.776/0.626	-
Initial model used	No model	ModelAngelo	AlphaFold3	ModelAngelo	No model	No model	AlphaFold3
Atomic Refinement package		PHENIX	PHENIX	PHENIX			PHENIX
Model resolution (Å)		2.8	7.5	3.5			7.6
FSC threshold		0.5	0.5	0.5			0.5
CCvolume/CCmask		0.86/0.88	0.71/0.72	0.81/0.82			0.59/0.58
Map sharpening *B* factor (Å^2)	0	+11	-127	-30	+50	+200	-62
Non-hydrogen atoms		14,940	9,410	11,554			9,662
Protein residues		1,900	1,894	1,435			1,948
Ligands		2xZn^{2+}	-	-			-
B factors (Å^2)							
Protein		70.6	333	53.1			96.3
Ligand		104	-	-			-
r.m.s.d.							
Bond lengths (Å)		0.003	0.024	0.004			0.002
Bond angles (°)		0.529	1.163	0.662			0.576
MolProbity score		1.44	1.13	2.11			0.65
Clashscore		3.24	1.34	8.33			0.43
Poor rotamers (%)		1.50	0	2.90			0
Ramachandran							
Favored (%)		96.85	96.12	95.62			98.51
Allowed (%)		3.15	3.67	4.31			1.49
Disallowed (%)		0	0.22	0.07			0
CaBLAM outliers (%)		1.52	1.21	2.37			0.22
EMRinger Score		2.80	*	1.53			*
Average Q-score		0.591	0.277	0.487			0.256

Table S4 – Cryo-EM data collection and refinement statistics (continued). See Figs. S14 and S17 for details. *Models did not include sidechains and so no EMRinger score could be calculated. 3DFSC sphericity values and estimated accuracy of rotations/offsets are only shown for maps refined in Relion (all apart from the AtUBR4 complex).

Rank	Protein	Peak area
	KCMF1	7.40E+09
	UBR4	4.77E+09
1	A0A2H4WW89_TRINI Tubulin alpha chain	1.19E+09
2	A0A7E5VJV7_TRINI tubulin beta-1 chain	7.49E+08
3	A0A7E5X521_TRINI 60S ribosomal protein L27a	5.01E+08
4	A0A7E5WXJ8_TRINI ubiquitin-40S ribosomal protein S27a	4.77E+08
5	A0A7E5WJQ8_TRINI 40S ribosomal protein S6	4.76E+08
6	A0A7E5WFJ6_TRINI 40S ribosomal protein S9	3.99E+08
7	A0A7E5VDL2_TRINI 40S ribosomal protein S8	3.40E+08
8	A0A7E5WBC0_TRINI 40S ribosomal protein S14	3.23E+08
9	A0A7E5WYR8_TRINI 40S ribosomal protein S2	2.80E+08
10	A0A7E5WY23_TRINI Heat shock protein 70	2.75E+08
11	A0A7E5WJR6_TRINI 40S ribosomal protein S3	2.65E+08
12	A0A7E5W5J4_TRINI 40S ribosomal protein S11 isoform X2	2.59E+08
13	A0A7E5WJV7_TRINI 60S ribosomal protein L17	2.53E+08
14	A0A7E5W4Q2_TRINI 40S ribosomal protein S27	2.46E+08
15	A0A7E5W8Z9_TRINI 40S ribosomal protein S16 isoform X1	2.13E+08
16	A0A2H4WW82_TRINI 40S ribosomal protein S18	1.85E+08
17	A0A7E5VWI8_TRINI eukaryotic translation initiation factor 3 subunit K	1.74E+08
18	A0A7E5WBC6_TRINI 60S ribosomal protein L24	1.66E+08
19	Q4VM05_TRINI 40S ribosomal protein S5	1.51E+08
20	A0A7E5W2S7_TRINI 40S ribosomal protein S3a	1.36E+08
21	A0A7E5WVI9_TRINI 60S ribosomal protein L32	1.36E+08
22	A0A7E5X5U8_TRINI 60S ribosomal protein L4	1.26E+08
23	A0A7E5VTX2_TRINI 60S ribosomal protein L19	1.20E+08
24	A0A7E5X3M5_TRINI 40S ribosomal protein S10	1.15E+08
25	A0A7E5WLS0_TRINI 40S ribosomal protein S26	1.11E+08
26	A0A7E5VNH5_TRINI 40S ribosomal protein S4	9.46E+07

27	A0A7E5X4C0_TRINI 60S ribosomal protein L21	8.94E+07
28	A0A7E5VYY3_TRINI 60S ribosomal protein L9	7.95E+07
29	A0A7G9U7K2_TRINI Heat shock protein 21.4	7.53E+07
30	A0A7E5W0B5_TRINI 60S ribosomal protein L18a	6.65E+07
31	A0A7E5X019_TRINI 60S ribosomal protein L13a	5.67E+07
32	A0A7E5VKI7_TRINI 40S ribosomal protein S23	5.39E+07
33	A0A7E5W784_TRINI 40S ribosomal protein S13	4.65E+07
34	A0A7E5WY71_TRINI 40S ribosomal protein S15Aa	4.33E+07
35	A0A7E5WEG5_TRINI 60S ribosomal protein L35	4.27E+07
36	A0A7E5WQP8_TRINI 60S ribosomal protein L10	4.13E+07
37	A0A7E5VLP1_TRINI 40S ribosomal protein S24	3.81E+07
38	A0A7E5X2F1_TRINI probable medium-chain specific acyl-CoA dehydrogenase, mitochondrial isoform X3	3.79E+07
39	A0A7E5X5Y1_TRINI tubulin beta chain-like	3.34E+07
40	A0A7E5WJ27_TRINI 60S ribosomal protein L6	3.32E+07
41	A0A7E5VE68_TRINI 60S ribosomal protein L35a	3.17E+07
42	A0A7E5WAV9_TRINI dnaJ homolog subfamily A member 2-like	3.13E+07
43	A0A7E5WF28_TRINI 60S ribosomal protein L27-like	2.83E+07
44	A0A7E5VSK5_TRINI heat shock protein 83	2.79E+07
45	A0A7E5VV98_TRINI 60S ribosomal protein L13	2.77E+07
46	A0A7E5X1Z1_TRINI 60S ribosomal protein L39	2.77E+07
	CALM1	2.64E+07
47	A0A7E5WUF4_TRINI 60S ribosomal protein L26	2.60E+07
48	A0A7E5WME8_TRINI 60S ribosomal protein L34-like	2.52E+07
49	A0A7E5X262_TRINI single-stranded DNA-binding protein, mitochondrial	2.17E+07
50	A0A7E5WNQ1_TRINI 60S ribosomal protein L23	1.97E+07

Table S5 – MS analysis of *Trichoplusia ni* contaminants in the purified CeUBR4 complex. Mitochondrial proteins are colored in red, while common contaminants are colored in blue (ribosome), orange (tubulin) and purple (chaperones).
